# How did the COVID-19 pandemic affect access to condoms, chlamydia and HIV testing, and cervical cancer screening at a population level in Britain? (Natsal-COVID)

**DOI:** 10.1136/sextrans-2022-055516

**Published:** 2022-08-18

**Authors:** Emily Dema, Pam Sonnenberg, Jo Gibbs, Anne Conolly, Malachi Willis, Julie Riddell, Raquel Bosó Pérez, Andrew J Copas, Clare Tanton, Chris Bonell, Clarissa Oeser, Soazig Clifton, Magnus Unemo, Catherine H Mercer, Kirstin R Mitchell, Nigel Field

**Affiliations:** 1 Institute for Global Health, University College London, London, UK; 2 Health and Biomedical Surveys, NatCen Social Research, London, UK; 3 Social and Public Health Sciences Unit, University of Glasgow MRC/CSO, Glasgow, UK; 4 Faculty of Public Health and Policy, London School of Hygiene and Tropical Medicine, London, UK; 5 Department of Laboratory Medicine, Örebro University Hospital, Orebro, Sweden

**Keywords:** COVID-19, SEXUAL HEALTH, CONDOMS, HIV, Diagnostic Screening Programs

## Abstract

**Objectives:**

To investigate how differential access to key interventions to reduce STIs, HIV and their sequelae changed during the COVID-19 pandemic.

**Methods:**

British participants (18–59 years) completed a cross-sectional web survey 1 year (March–April 2021) after the initial lockdown in Britain. Quota-based sampling and weighting resulted in a quasi-representative population sample. We compared Natsal-COVID data with Natsal-3, a household-based probability sample cross-sectional survey (16–74 years) conducted in 2010–2012. Reported unmet need for condoms because of the pandemic and uptake of chlamydia testing/HIV testing/cervical cancer screening were analysed among sexually experienced participants (18–44 years) (n=3869, Natsal-COVID; n=8551, Natsal-3). ORs adjusted for age and other potential confounders describe associations with demographic and behavioural factors.

**Results:**

In 2021, 6.9% of women and 16.2% of men reported unmet need for condoms because of the pandemic. This was more likely among participants: aged 18–24 years, of black or black British ethnicity, and reporting same-sex sex (past 5 years) or one or more new relationships (past year). Chlamydia and HIV testing were more commonly reported by younger participants, those reporting condomless sex with new sexual partners and men reporting same-sex partners; a very similar distribution to 10 years previously (Natsal-3). However, there were differences during the pandemic, including stronger associations with chlamydia testing for men reporting same-sex partners; with HIV testing for women reporting new sexual partners and with cervical screening among smokers.

**Conclusions:**

Our study suggests differential access to key primary and secondary STI/HIV prevention interventions continued during the first year of the COVID-19 pandemic. However, there was not strong evidence that differential access has changed during the pandemic when compared with 2010–2012. While the pandemic might not have exacerbated inequalities in access to primary and secondary prevention, it is clear that large inequalities persisted, typically among those at greatest STI/HIV risk.

**WHAT IS ALREADY KNOWN ON THIS TOPIC**

- Though the pandemic disrupted sexual behaviour and sexual and reproductive health (SRH) services, it is unknown how pre-existing disparities in STI/HIV prevention were affected.

**WHAT THIS STUDY ADDS**

- This study compared differential access to key SRH interventions using Natsal-COVID (2021) and Natsal-3 (2010–2012) data. Many men who have sex with men, people of black ethnicity and young people reported unmet need for condoms because of the pandemic, but there was not strong evidence that these key populations were at additional risk during the pandemic compared with 2010–2012.

**HOW THIS STUDY MIGHT AFFECT RESEARCH, PRACTICE OR POLICY**

- Improving accessibility to free or low-cost condoms in Britain should be prioritised. Large inequalities in access to key STI/HIV interventions persist, and there remains a need to reduce, if not eradicate, these.

## Introduction

Primary and secondary prevention methods interrupt the transmission or consequences of STIs and HIV. For STIs/HIV, primary prevention aims to prevent infection occurring at all (eg, condoms), while secondary prevention involves detection/treatment of infection before disease manifestations (eg, testing for and treating early chlamydia or HIV infection, or cervical cancer screening to detect abnormal cells and cervical intraepithelial neoplasia caused by infection with high-risk human papillomavirus).^1^ Such interventions remained important during the COVID-19 pandemic because potentially risky sexual activity continued despite lockdowns,^2^ and STI/HIV diagnoses nearly regained pre-pandemic levels by the end of 2020.^3^ Different population groups experienced significant health inequalities during the pandemic due to the direct impacts of COVID-19, as well as impacts on the wider health system and society.^4^ There were also significant pre-existing inequalities in uptake of sexual and reproductive health (SRH) interventions and outcomes,^5–7^ and the pandemic disrupted SRH services, which likely delayed diagnoses and led to worse outcomes. However, it is unknown whether or how the pandemic affected inequalities in STI/HIV prevention.

In Britain, a national lockdown was announced on 23 March 2020, which lasted approximately 4 months and caused the most severe disruption. Restrictions continued throughout 2020. Another 4-month national lockdown began in early January 2021. During this period, SRH services were impacted by reduced face-to-face consultations and the need to prioritise key populations and symptomatic patients, as well as by concerns about the risk of SARS-CoV-2 infection, which affected health-seeking behaviour.^8 9^

The National Survey of Sexual Attitudes and Lifestyles (Natsal)-COVID web-panel study was conducted to understand the population-level impact of the COVID-19 pandemic on SRH in Britain. Survey Wave 1 of Natsal-COVID was conducted 4 months (July–August 2020) after the announcement of the first national lockdown to understand initial changes in SRH service use.^10 11^ STI services were most likely to reach those most at risk of STIs in those first 4 months, though there were often difficulties in access.^10 12^ Survey Wave 2, conducted a year after the initial lockdown, captured key annual STI outcomes, such as HIV and chlamydia testing.^13^ Elsewhere, we have reported an overall reduction in chlamydia testing for Wave 2 compared with Natsal-3 (a household-based representative probability sample survey of the British population conducted from 2010 to 2012), while HIV testing and STI-related service use were similar to Natsal-3.^14^

In this paper, we investigated whether and how underlying differential access to key STI/HIV interventions by population group changed during the first year of the pandemic. We used Natsal-COVID survey Wave 2 data on reported unmet need for condoms, chlamydia and HIV testing, and cervical cancer screening to assess the distribution in the general population and among key populations experiencing a disproportionate burden of diagnoses (including men who have sex with men (MSM), young people and people of black ethnicity).^15^ We compared these distributions with data from Natsal-3 (2010–2012) as the most recent representative population survey on sexual health in Britain. We hypothesised that differential access to key STI interventions was exacerbated due to the pandemic.

## Methods

### Natsal-COVID Wave 2 study design

Natsal-COVID survey Wave 2 was a quasi-representative web-panel survey of sexual health conducted 1 year after the first national lockdown in Britain. Data were collected using a short online questionnaire (median completion time: 13 min) through survey research company Ipsos-MORI’s web panel. Participants were asked about uptake of STI interventions in the 1 year from 23 March 2020. The sample comprised longitudinal participants, who completed Wave 1, and new cross-sectional participants recruited at Wave 2. The questionnaire is available at https://www.natsal.ac.uk/natsal-covid-study. Details of the Natsal-COVID methods are described elsewhere.^13^

### Participants and procedures of Natsal-COVID Wave 2

Altogether, 6658 participants completed the survey between 27 March and 26 April 2021, including 2098 who also participated in Wave 1. To achieve a quasi-representative sample of the British population, we used quotas for age, gender, region (based on Office for National Statistics 2019 midyear estimates) and social grade (based on Census 2011 data), and weighted the data to match the general population distributions for the quotas, ethnicity and sexual identity. An anonymised dataset will be deposited with the UK Data Service to accompany the Natsal-COVID survey Wave 1 data (SN8865) and datasets from previous decennial Natsal surveys, including Natsal-3 (SN7799).

### Comparison with Natsal-3

We compared our findings with data from the Natsal-3 survey. Natsal-3 (2010–2012) used a multistage, clustered and stratified probability sample design.^16^ Interviewers visited all sampled addresses, identified residents in the eligible age range (16–74 years) and randomly selected one individual to participate. Participants then completed the survey in their own homes through a combination of face-to-face interviews and a self-completion interview. Interviews lasted about 1 hour on average. Details of the Natsal-3 methods are described elsewhere.^16^

### Statistical measures and analysis

We used Stata (V.16.1) complex survey analysis functions to incorporate weighting and stratification. Outcomes of interest are shown in online supplemental table 1.

10.1136/sextrans-2022-055516.supp1 Supplementary data

Data from Natsal-COVID are presented for all participants and separately for men (including trans men) and women (including trans women). While we did not present estimates for participants who identified ‘in another way’, these 22 participants were included in estimates presented for ‘all’. For analysis of cervical cancer screening, we included all participants described female at birth, which included some trans men and non-binary people. Natsal-3 used a binary measure of gender.

We examined the outcome of ‘unmet need for condoms’ among sexually experienced participants (ie, any lifetime vaginal, anal, oral sex or other genital contact) by asking ‘Was there any time since the start of the first lockdown when you needed to use condoms, but didn’t because you couldn’t get hold of any because of the pandemic?’ Participants aged 45–59 years were excluded due to low burden of STIs in this age group. Of 6658 Natsal-COVID participants aged 18–59 years, 4323 were aged 18–44 years, and 3869 were sexually experienced and included in analysis. Although some sexually experienced participants (n=270 men and n=240 women) did not report sexual partners in the past year, they were included in denominators for ‘unmet need for condoms’ since disrupted access to condoms might have prevented some participants from having sex. This question was not asked in Natsal-3.

We estimated reported chlamydia and HIV testing in the past year among sexually experienced participants (18–44 years) for Natsal-COVID and Natsal-3. Natsal-3 participants reporting at least one lifetime sexual partner were considered ‘sexually experienced’. Of 15 162 Natsal-3 participants, 8969 were aged 18–44 years, and 8551 were sexually experienced and included in analysis.

We estimated reported cervical cancer screening among eligible participants (ie, reported being described female at birth (Natsal-COVID) or women (Natsal-3) and aged 25–59 years). This age group was chosen to closely reflect UK national screening programme eligibility (25–64 years). Cervical screening estimates are presented for eligible participants for the past year (Natsal-COVID) or past 3 years (Natsal-3); therefore, we focused on comparing characteristics associated with the uptake of cervical screening between surveys, rather than prevalence estimates.

MSM in Natsal-COVID and Natsal-3 were defined as men (based on reported gender identity in Natsal-COVID) reporting at least one same-sex partner (defined by participant) in the past 5 years.

We used logistic regression to calculate age-adjusted ORs (aORs) to investigate how uptake varied by sociodemographic and behavioural factors. To establish independent associations with ‘unmet need for condoms’, the model was also adjusted for sociodemographic (age, region, rurality, ethnicity and relationship formation) and behavioural (sexual partners in the past year and previous same-sex experience in the past 5 years) factors. Where possible, we compared aORs in Natsal-COVID analyses with those generated from Natsal-3 data to investigate whether and how patterns of association differed between these studies. We describe the differences in the strength of associations and test for differences in the distribution of associations by including interaction terms in the regression models.

### Patient and public involvement

Patients or the public were not directly involved in the design, conduct, reporting or dissemination plans of the Natsal-COVID Study due to the urgency of the research during the pandemic. However, members of the public were involved in the design of the Natsal-4 questionnaire, upon which the Natsal-COVID questionnaire was based.

## Results

### Primary STI prevention

#### Unmet need for condoms

Among sexually experienced participants (18–44 years), 6.9% of women and 16.2% of men reported unmet need for condoms in the past year because of the pandemic (online supplemental table 2). Participants aged 18–24 years (women 16.8% and men 33.1%) and MSM (36.8%) were more likely to report this. Unmet need was even higher in young MSM (50.4% of 89 MSM aged 18–29 years old).

In an adjusted model, unmet need for condoms was most likely to be reported by younger participants and, among men, those identifying as black or black British (online supplemental table 2). Participants who reported symptoms of depression or anxiety were also more likely to report unmet need.

There were strong associations between unmet need for condoms and behavioural markers of HIV/STI risk. Participants who reported forming new relationships in the past year or a same-sex experience in the past 5 years were more likely to report unmet need (44.1% of women who reported previous same-sex experience also reported at least one opposite sex partner in the past 5 years). Among participants who reported unmet need, 47.0% (39.6%–54.5%) of men and 34.4% (25.9%–44.0%) of women also reported condomless sex on the first occasion with a new partner during the past year. By comparison, in the group that did not report unmet need, only 13.9% (11.7%–16.4%) of men and 8.6% (7.3%–10.2%) of women reported condomless sex on the first occasion with a new partner (aOR for condomless with new partner: women, 4.42 (2.81–6.95); men, 4.67 (3.21–6.78); data not shown). Among men but not women, participants who reported use of STI-related services in the past year were more likely to report unmet need in the adjusted model.

### Secondary STI prevention

#### Chlamydia and HIV testing

Among sexually experienced participants (18–44 years), 7.3% of women and 4.1% of men reported a chlamydia test in the past year, which was significantly lower than the proportions reported in Natsal-3 (2010–2012) (25.1% women; 15.1% men). HIV testing in the past year was reported by 8.6% of women and 6.5% of men in Natsal-COVID Wave 2, similar to the 10.4% of women and 6.0% of men in Natsal-3 (online supplemental tables 3 and 4).

**Figure 1:**
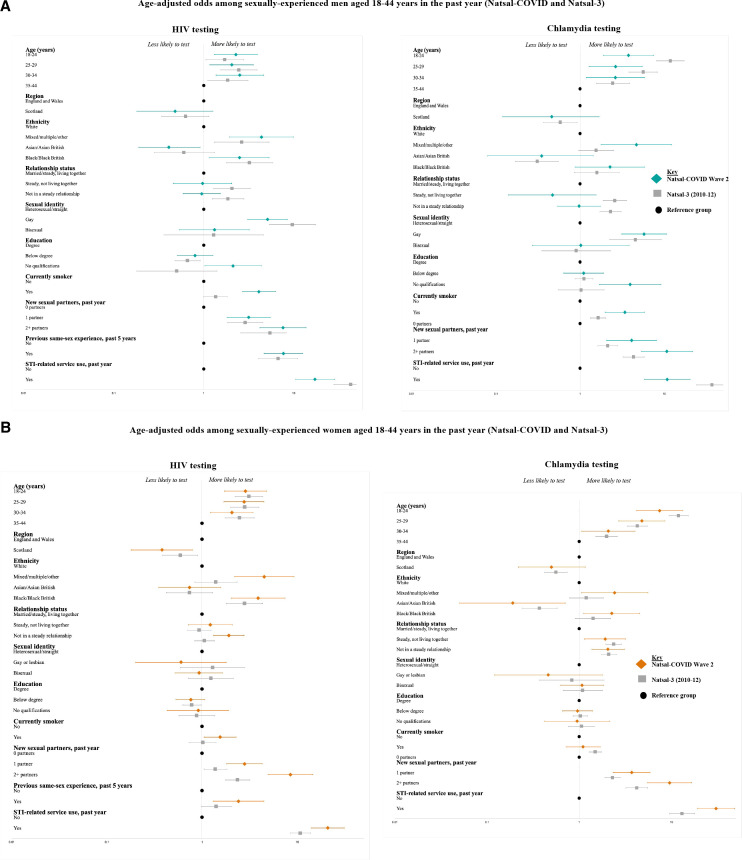
(A) Age-adjusted odds among sexually experienced men aged 18-44 years in the past year (Natsal-COVID and Natsal-3). (B) Age-adjusted odds among sexually experienced women aged 18-44 years in the past year (Natsal-COVID and Natsal-3). Natsal, National Survey of Sexual Attitudes and Lifestyles.

The direction and strength of associations for most independent variables with chlamydia and HIV testing were similar for Natsal-COVID and Natsal-3, based on interaction terms (figure 1, online supplemental tables 3 and 4). In both surveys, participants aged 18–24 years were more likely to report an HIV test compared with those aged 35–44 years; black or black British participants were more likely to report testing than white participants, and MSM were more likely than other men to report testing. In each case, the strength of associations was similar.

Nevertheless, there were some statistically significant interactions suggesting several differences between tsurveys. For example, young people (18–24 years) were significantly more likely to report chlamydia testing compared with the oldest age group in both surveys, and while the strength of this age association was similar for women across surveys, it was significantly stronger for men in Natsal-3 than Natsal-COVID (interaction p=0.01). MSM were more likely to report chlamydia testing in Natsal-COVID than Natsal-3 (interaction p=0.04).

#### Cervical cancer screening

Among eligible participants in Natsal-COVID, 10.3% reported use of cervical cancer screening services in the past year. In Natsal-3, 70.6% of women reported cervical screening in the past 3 years.

**Figure 2:**
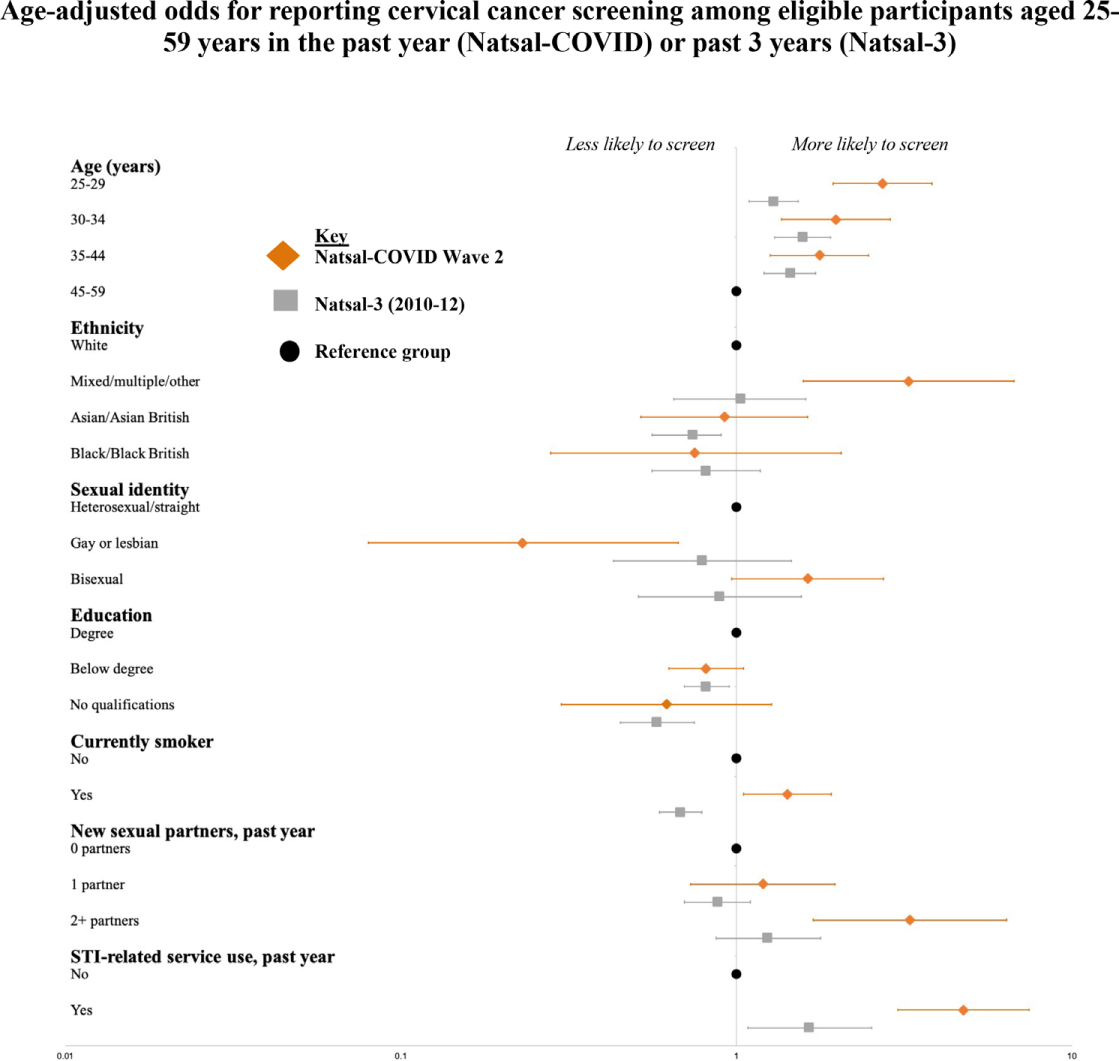
Age-adjusted odds for reporting cervical cancer screening among eligible participants aged 25–59 years in the past year (Natsal-COVID) or past 3 years (Natsal-3). Natsal, National Survey of Sexual Attitudes and Lifestyles.

Associations for reported cervical screening were broadly similar to those in Natsal-3 (figure 2, online supplemental table 5). The youngest participants (25–29 years) were more likely to report screening compared with participants aged 44–59 years in both surveys, although the association with age was stronger in Natsal-COVID than Natsal-3 (interaction p=0.01). Gay or lesbian participants were less likely to screen than heterosexual participants in Natsal-COVID, while there was no association with sexual identity in Natsal-3 (interaction p=0.01). Notably, participants who reported smoking were more likely to report screening in Natsal-COVID, while this same group was less likely to screen in Natsal-3. Cervical screening was also associated with markers of sexual risk, such as reporting two or more sexual partners in the past year, inNatsal-COVID but not Natsal-3 (interaction p=0.01).

## Discussion

### Principal findings

Findings from this large, quasi-representative survey of the British population indicate differential access to key STI/HIV prevention interventions during the COVID-19 pandemic, particularly for young people, MSM and those reporting new sexual partners. However, we did not find strong evidence that differential access for these key populations had changed during the pandemic when compared with 2010–2012.

Regarding primary prevention, use of condoms is a highly cost-effective way to prevent transmission of STIs/HIV and unplanned pregnancy.^17^ However, 6.9% of women and 16.2% of men aged 18–44 years reported unmet need for condoms in the past year because of the pandemic. This was even higher for young men aged 18–24 years (33%) and MSM aged 18–29 years (50%). Participants who reported one or no partners in the past year (ie, low STI risk) still reported unmet need, which could indicate that some people were avoiding sex because they were unable to access condoms. It is also striking that participants reporting symptoms of depression or anxiety were more likely to report unmet need, though we are unable to determine causality. On the other hand, participants who reported unmet need were more likely to report sexual behaviours associated with STI/HIV risk. For example, they were more likely to report condomless sex with new partners, which suggests that improving access to condoms might support higher levels of condom use with new partners, in turn reducing STI/HIV transmission. Notably, many men reporting unmet need also reported use of STI-related services in the past year, suggesting a role for SRH services in improving access to free or low-cost and easily accessible condoms. Anecdotal evidence suggests that provision of condoms at SRH services has reduced in the past decade and that remote service provision has further limited access during the pandemic. MSM, people of black ethnicity and young people are among the groups most impacted by STIs in Britain,^15^ and it is concerning that a high proportion of individuals in these key populations were unable to access condoms when they needed them. A Scottish web survey conducted in July 2020 corroborates our findings on unmet need for condoms, especially among young people.^18^ Our data suggest that improving accessibility to free or low-cost condoms should be prioritised.

The distribution in the population of reporting chlamydia and HIV testing was broadly similar for Natsal-COVID (2021) and Natsal-3 (2010–2012). Key populations at most risk of STI transmission, including young people, MSM and those reporting condomless sex with new partners, continue to be most likely to engage with SRH services, and the strengths of association between the different groups were similar in both surveys. In the past decade, HIV testing among MSM has increased due to targeted campaigns.^19^ However, we did not detect a stronger association with HIV testing among MSM in Natsal-COVID compared with Natsal-3—potentially due to a reversal of the upward trend in HIV testing among MSM in the years immediately prior to the pandemic.^19 20^

Although we cannot compare population estimates because of differences in the reporting time frames, patterns of reported access to cervical cancer screening were similar in Natsal-COVID and Natsal-3. However, there was higher reported use among younger participants (25–29 years) in Natsal-COVID, which might suggest either a longer-term trend over the past decade and/or a greater willingness to access services during the pandemic in younger compared with older participants, who might have perceived higher risk of severe COVID-19. In Natsal-3, reported uptake of cervical cancer screening was lower among smokers, while this group was more likely to screen in Natsal-COVID. At a population level, smoking has declined substantially in the past decade, particularly among those aged 18–24 years old.^21^ Nevertheless, that smokers were more likely to report cervical screening could be positive, given the additional risk for cervical cancer brought by smoking.^22^ Surveillance data suggest a decrease in invitations and screening in 2020 compared with 2019, which corroborates Natsal-COVID Wave 1 and ave 2 findings suggesting a potential backlog of need for cervical screening.^10 14 23^

### Comparison with other studies

Reprioritisation of healthcare services, including SRH, due to COVID-19 led to unmet need,^10^ even though there was a reduction in new partners, particularly among young people and MSM.^14^ Data from the UK Health Security Agency demonstrated a fall in bacterial STI testing from 2019 to 2020 among younger people, people of Asian or black ethnicity, and heterosexual men, though there was a small increase in testing among MSM.^24^ Surveillance data also showed the burden of STIs remained greatest in those aged 15–24 years, as well as black ethnic minorities and MSM in 2020.^15^

### Strengths and limitations

No previous study has examined whether and how differential access to key interventions to prevent STI or HIV and their sequelae changed at a population level due to the COVID-19 pandemic.^20^ Missing data were low for Natsal-COVID (ie, non-response was 1%–4%). However, our study also has limitations.^13^ While benefiting from a questionnaire developed by the Natsal team to obtain high-quality data while navigating pandemic-related circumstances and using a large national sample, with quota sampling and weighting to improve generalisability, the Natsal-COVID study is not a probability sample. Specific prevalence estimates should be treated withcaution given expected selection and response biases. The question on ‘unmet need for condoms’ was not validated due to time constraints on questionnaire development.

Due to the lack of population-level data on key STI/HIV prevention intervention access by sociodemographic and behavioural characteristics collected immediately prior to the pandemic, we used data from Natsal-3 to compare trends in differential access, which serves as a proxy for inequalities in access. Natsal-3 data provided the best comparison for these population-level STI/HIV interventions—with four key caveats. First, Natsal-3 data were collected 10 years ago, so sexual behaviours and service provision have likely undergone secular changes since then. Second, there are different sampling biases between the surveys that weighting can only partially correct.^13^ Third, it was not possible to determine whether differences in associations were because of a change in the risk group, or a change in the reference group (or both). Likewise, where there was no difference between the surveys, this might be due to methodological differences. Finally, it is not clear whether differences with Natsal-3 are pandemic related or indicative of longer-term secular trends. Therefore, while the associations in the Natsal-COVID are strikingly similar to Natsal-3, comparisons should be interpreted with caution.

### Conclusions and policy implications

Our study suggests differential access to key STI/HIV prevention interventions during the first year of the COVID-19 pandemic. However, available evidence does not suggest substantial changes in the patterns of uptake since 2010–2012. While the pandemic might not have exacerbated inequalities in access, we did observe that large inequalities persist. These were typically among those at greatest STI/HIV risk, and there remains a need to reduce, if not eradicate, these. Future comparison with the fourth decennial probability survey (Natsal-4), which starts fieldwork in 2022, will be critical to continue to monitor inequalities and trends more broadly.

## Data Availability

Data are available in a public, open access repository. An anonymised dataset will be deposited with the UK Data Archive.
