# Supplementary material for: How did the COVID-19 pandemic affect access to condoms, chlamydia and HIV testing, and cervical cancer screening at a population level in Britain? (Natsal-COVID)
Source: Sex Transm Infect. 2022 Aug 18;99(4):261–7. doi: 10.1136/sextrans-2022-055516 (PMC10313967; doi:10.1136/sextrans-2022-055516)
Supplement: Supplementary data [file sextrans-2022-055516supp001.pdf]

Supplementary Material

Supplementary table 1. Outcomes of interest in Natsal-COVID and Natsal-3

Outcome of interest	Natsal-COVID	Natsal-3	Denominator	Timeframe
Unmet need for condoms (Natsal-COVID only)	Was there any time since the start of the first lockdown when you needed to use condoms, but didn't because you couldn't get hold of any because of the pandemic?<br>**1. Yes**<br>2. No<br>3. Prefer not to say	N/A	Sexually experienced participants aged 18-44y	Past year
Chlamydia testing	Have you ever been tested for chlamydia?<br>**1. Yes**<br>2. No<br>3. Not sure<br>4. Prefer not to say<br>[If 'Yes' then asked] When were you last tested for chlamydia? Please think about your last chlamydia test, whatever the result.<br>**1. In the last year**<br>2. Between 1 and 5 years ago<br>3. More than 5 years ago<br>4. Not sure	In the last year, have you been tested for Chlamydia?<br>**1. Yes**<br>2. No	Sexually experienced participants aged 18-44y	Past year

	5. Prefer not to say			
HIV testing	Have you ever had a test for HIV?<br>**1. Yes**<br>2. No<br>3. Not sure<br>4. Prefer not to say<br><br>[If 'Yes' then asked] When was your most recent HIV test?<br>**1. In the last three months**<br>**2. Between 3 months and 1 year ago**<br>3. Between 1 and 5 years ago<br>4. More than 5 years ago<br>5. Prefer not to say	Have you ever had a test for HIV (the virus that causes AIDS)?<br>**1. Yes**<br>2. No<br>3. Maybe/not sure<br><br>[If 'Yes' then asked] When was that test? (the last HIV test if more than one)<br>**1. In the last year**<br>2. Between 1 and 2 years ago<br>3. Between 2 and 5 years ago<br>4. Longer than 5 years ago	Sexually experienced participants aged 18-44y	Past year
Cervical cancer screening	Since the start of the first lockdown (23 March 2020), did you use any of the following sexual or reproductive health services for yourself? Please include phone, online or video appointments.<br>1. None<br>2. Contraception services/advice<br>3. Fertility services/advice	When did you last have a cervical smear test?<br>1. I have never had one<br>**2. Less than 3 years ago**<br>3. Between 3 and 5 years ago	Eligible participants aged 25-59y. Natsal-3 used a binary measure of gender, while Natsal-COVID asked about sex at birth and gender identity. Analysis of cervical screening data was limited to just women in Natsal-3, but included women and trans participants in Natsal-COVID	Past year (Natsal-COVID) or past three years (Natsal-3)

	4. Maternity/antenatal services<br>5. Abortion/Pregnancy termination services<br>**6. Cervical screening (smear test/pap test)**<br>7. STI (Sexually Transmitted Infection) testing<br>8. STI follow-up care<br>9. HIV testing<br>10. Advice or counselling for sexual problems<br>11. Relationship support services/advice<br>12. Sexual assault/rape support services or helplines<br>13. Other type of sexual or reproductive health service/advice<br>14. Prefer not to say	4. Between 5 and 10 years ago<br><br>5. More than 10 years ago		

Supplementary table 2. Variations in reporting unmet need for condoms because of the pandemic among sexually-experienced women and men aged 18-44 years in the first year following the start of a national lockdown in Britain (23/03/2020)

	**Women (sexually-experienced)**											**Men (sexually-experienced)**										
	**Weighted %**	**95% CI**	**OR**	**95% CI**	**aOR***	**95% CI**	**Model 1 AOR****	**95% CI**	**Model 2 AOR*****	**95% CI**	**Denominator † (unweighted, weighted)**	**Weighted %**	**95% CI**	**OR**	**95% CI**	**aOR***	**95% CI**	**Model 1 AOR****	**95% CI**	**Model 2 AOR*****	**95%CI**	**Denominator † (unweighted, weighted)**
**All ages (18-44 years)**	6.9%	[5.8%,8.3%]	-	-	-	-	-	-	-	-	1997, 1683	16.2%	[14.2%, 18.4%]	-	-	-	-	-	-	-	-	1511, 1686

**Age (years)** **,***			**p<0.001**				**p=0.0046**		**p=0.02**					**p<0.001**				**p<0.001**		**p=0.005**		
18-24	16.8%	[12.8%, 21.8%]	5.96	(3.49-10.18)	-	-	3.03	(1.50-6.12)	2.95	(1.42-6.16)	371, 290	33.1%	[27.3%, 39.4%]	6.51	(4.27-9.91)	-	-	2.18	(1.23-3.86)	2.25	(1.26-4.01)	307, 342
25-29	7.7%	[5.5%,10.8%]	2.48	(1.41-4.35)	-	-	2.19	(1.17-4.07)	2.28	(1.18-4.40)	518, 416	23.0%	[18.2%, 28.6%]	3.92	(2.54-6.05)	-	-	2.59	(1.57-4.27)	2.30	(1.35-3.92)	326, 336
30-34	4.4%	[2.7%,7.1%]	1.36	(0.70-2.65)	-	-	1.03	(0.47-2.24)	1.27	(0.57-2.79)	402, 330	11.5%	[7.8%,16.7%]	1.71	(1.00-2.92)	-	-	1,15	(0.58-2.29)	1.19	(0.59-2.39)	226, 264
35-44	3.3%	[2.2%,4.9%]	1.00		-	-	1.00		1.00		706, 647	7.1%	[5.2%,9.5%]	1.00		-	-	1.00		1.00		652, 744
**Region** **,***			**p=0.04**		**p=0.0310**		**p=0.1715**		**p=0.24**					**p=0.03**		**p=0.02**		**p=0.27**		**p=0.36**		
England/Wales	7.4%	[6.1%,8.8%]	1.00		1.00		1.00		1.00		1820, 1520	17.0%	[14.9%, 19.3%]	1.00		1.00		1.00		1.00		1382, 1534
Scotland	3.0%	[1.2%,6.9%]	0.38	(0.15-0.96)	0.36	(0.14-0.91)	0.49	(0.18-1.36)	0.55	(0.20-1.50)	177, 163	8.0%	[4.0%,15.5%]	0.43	(0.20-0.91)	0.38	(0.17-0.84)	0.58	(0.22-1.53)	0.64	(0.24-1.69)	129, 152
**Rurality** **,***			**p=0.01**		**p=0.0141**		**p=0.0613**		**p=0.05**					**p=0.03**		**p=0.04**		**p=0.59**		**p=0.56**		
Urban	7.6%	[6.2%,9.3%]	1.00		1.00		1.00		1.00		1406, 1184	16.2%	[14.0%, 18.7%]	1.00		1.00		1.00		1.00		1138, 1271
Rural	3.0%	[1.5%,6.0%]	0.38	(0.18-0.8	0.35	(0.15-0.8	0.47	(0.21-1.0	0.41	(0.16-1.0	247, 199	8.7%	[4.9%,15.1%]	0.49	(0.26-0.9	0.49	(0.25-0.9	0.83	(0.41-1.6	0.81	(0.40-1.6	131, 153

				0)		1)		4)		1)					4)		7)		5)		6)	
**Ethnicity** **, ***			**p<0.001**		**p<0.001**		**p=0.0076**		**p=0.007**					**p<0.001**		**p<0.001**		**p=0.02**		**p=0.02**		
White [1]	5.1%	[4.1%,6.4%]	1.00		1.00		1.00		1.00		1736, 1408	13.4 %	[11.4%, 15.6%]	1.00		1.00		1.00		1.00		1253, 1381
Mixed, multiple, or other [2]	13.1 %	[6.5%,24.4%]	2.77	(1.25-6.16)	2.68	(1.23-5.81)	2.76	(0.91-8.43)	3.29	(1.10-9.85)	75, 57	34.2 %	[19.3%, 53.0%]	3.37	(1.52-7.47)	2.35	(1.04-5.28)	1.24	(0.45-3.43)	1.11	(0.39-3.18)	54, 69
Asian or Asian British [3]	14.9 %	[9.4%,22.9%]	3.24	(1.83-5.74)	3.04	(1.67-5.54)	2.62	(1.22-5.62)	2.77	(1.17-6.54)	116, 129	24.1 %	[16.6%, 33.6%]	2.06	(1.25-3.40)	1.74	(1.04-2.90)	1.29	(0.65-2.60)	1.54	(0.76-3.13)	120, 148
Black or Black British [4]	24.7 %	[14.2%, 39.4%]	6.05	(2.94-12.46)	4.43	(2.16-9.09)	2.81	(1.10-7.14)	1.93	(1.03-8.30)	52, 65	44.4 %	[31.2%, 58.4%]	5.17	(2.86-9.36)	3.73	(2.06-6.73)	3	(1.48-6.07)	2.86	(1.45-5.66)	56, 61
**Sexual identity**			**p=0.10**		**p=0.60**		**p=0.84**		**p=0.08**					**p=0.005**		**p=0.02**		**p=0.08**		**p<0.001**		
Heterosexual/straight	6.70 %	[5.6%,8.2%]	1.00		1.00		1.00		1.00		1714, 1594	16.20 %	[14.2%, 18.5%]	1.00		1.00		1.00		1.00		1298, 1596
Gay or lesbian	10.00 %	[4.4%,21.2%]	1.54	(0.62-3.81)	1.23	(0.48-3.12)	1.43	(0.46-4.46)	0.15	(0.03-0.74)	62, 19	4.90 %	[2.1%,11.1%]	0.27	(0.11-0.65)	0.23	(0.09-0.60)	0.24	(0.08-0.70)	0.06	(0.02-0.21)	117, 47
Bisexual	12.20 %	[7.7%,18.8%]	1.93	(1.11-3.33)	1.46	(0.82-2.58)	0.98	(0.44-2.19)	0.43	(0.16-1.14)	177, 42	31.10 %	[16.4%, 51.0%]	2.33	(1.00-5.44)	1.40	(0.60-3.28)	0.93	(0.40-2.15)	0.28	(0.09-0.87)	66, 19
Other	-	-	-	-	-	-	-	-	-	-	27, 11 ****	-	-	-	-	-	-	-	-	-	-	17, 10 ****

**Highest education qualification level**			**p=0.01**		**p=0.08**		**p=0.20**		**p=0.60**					**p<0.001**		**p<0.001**		**p=0.003**		**p=0.002**		
Degree	5.80%	[4.4%,7.6%]	1.00		1.00		1.00		1.00		1027, 869	14.40%	[11.8%, 17.4%]	1.00		1.00		1.00		1.00		750, 771
Below degree	7.30%	[5.6%,9.5%]	1.28	(0.85-1.92)	1.07	(0.70-1.62)	1.03	(0.61-1.72)	1.05	(0.61-1.79)	880, 736	15.20%	[12.5%, 18.4%]	1.07	(0.77-1.48)	0.85	(0.60-1.20)	0.95	(0.62-1.46)	1.10	(0.71-1.72)	676, 818
No qualifications	15.70%	[9.2%,25.3%]	3.00	(1.54-5.85)	2.28	(1.10-4.70)	2.10	(0.90-4.90)	1.71	(0.59-4.94)	90, 78	38.80%	[27.6%, 51.2%]	3.77	(2.17-6.57)	2.65	(1.47-4.78)	3.47	(1.66-7.26)	3.64	(1.78-7.45)	85, 98
**Days drinking, past 7 days**			**p<0.001**		**p<0.001**		**p=0.0002**		**p<0.001**					**p<0.001**		**p<0.001**		**p<0.001**		**p<0.001**		
0 days	3.70%	[2.5%,5.4%]	1.00		1.00		1.00		1.00		857, 730	8.90%	[6.2%,12.5%]	1.00		1.00		1.00		1.00		453, 496
1-2 days	6.80%	[5.1%,9.1%]	1.91	(1.15-3.16)	1.72	(1.02-2.88)	1.59	(0.86-2.95)	1.31	(0.70-2.47)	755, 630	16.10%	[13.1%, 19.6%]	1.96	(1.25-3.08)	1.89	(1.17-3.04)	2.06	(1.13-3.76)	2.05	(1.11-3.77)	606, 706
3-4 days	14.90%	[10.7%, 20.3%]	4.54	(2.61-7.89)	4.03	(2.30-7.06)	3.79	(1.92-7.49)	3.38	(1.67-6.84)	255, 218	25.30%	[20.2%, 31.3%]	3.47	(2.14-5.64)	3.92	(2.36-6.49)	3.80	(2.03-7.12)	3.67	(1.95-6.92)	299, 320
5-7 days	14.20%	[8.4%,22.9%]	4.30	(2.11-8.77)	4.99	(2.43-10.24)	3.98	(1.76-9.02)	3.61	(1.54-8.48)	126, 101	21.00%	[14.8%, 28.8%]	2.72	(1.54-4.81)	3.22	(1.82-5.71)	2.17	(1.06-4.46)	1.62	(0.72-3.65)	151, 162

**Currently smoker**			**p<0.001**		**p<0.001**		**p<0.001**		**p<0.001**					**p<0.001**		**p<0.001**		**p<0.001**		**p<0.001**		
No	4.20%	[3.2%,5.4%]	1.00		1.00		1.00		1.00		1541, 1301	10.40%	[8.5%,12.7%]	1.00		1.00		1.00		1.00		1027, 1128
Yes	16.30%	[12.8%, 20.4%]	4.47	(3.01-6.63)	4.39	(2.93-6.59)	3.87	(2.41-6.23)	3.15	(1.92-5.17)	447, 374	28.00%	[23.7%, 32.7%]	3.34	(2.43-4.58)	2.99	(2.16-4.15)	2.13	(1.44-3.15)	2.19	(1.45-3.30)	474, 549
**Importance of sexual health, past year**			**p=0.003**		**p=0.02**		**p=0.12**		**p=0.11**					**p<0.001**		**p=0.003**		**p=0.20**		**p=0.18**		
Very important/somewhat important	8.50%	[7.0%,10.4%]	1.00		1.00		1.00		1.00		1293, 1091	18.90%	[16.3%, 21.8%]	1.00		1.00		1.00		1.00		955, 1086
Not very important/not important	4.10%	[2.7%,6.3%]	0.46	(0.28-0.76)	0.53	(0.32-0.87)	0.58	(0.33-1.04)	0.52	(0.27-1.02)	553, 463	13.20%	[10.0%, 17.1%]	0.65	(0.46-0.93)	0.65	(0.45-0.94)	0.76	(0.48-1.21)	0.75	(0.47-1.21)	441, 478
This does not apply to me	2.80%	[0.7%,10.4%]	0.31	(0.07-1.28)	0.36	(0.09-1.48)	0.34	(0.04-2.61)	0.32	(0.04-2.62)	114, 98	3.10%	[1.0%,9.2%]	0.14	(0.04-0.44)	0.20	(0.06-0.64)	0.33	(0.08-1.45)	0.33	(0.08-1.35)	96, 101
**Symptoms of depression (PHQ-2) [7]**			**p<0.001**		**p<0.001**		**p=0.004**		**p=0.01**					**p<0.001**		**p<0.001**		**p<0.001**		**p<0.001**		
No	3.90%	[2.9%,5.3%]	1.00		1.00		1.00		1.00		1240, 1061	9.50%	[7.6%,11.8%]	1.00		1.00		1.00		1.00		938, 1046
Yes	11.20%	[8.9%,14.1%]	3.11	(2.05-4.7	2.53	(1.63-3.9	2.12	(1.27-3.5	1.99	(1.17-3.3	724, 596	26.40%	[22.5%, 30.8%]	3.43	(2.48-4.7	2.87	(2.04-4.0	2.46	(1.63-3.7	2.23	(1.46-3.4	548, 616

				1)		3)		6)		9)					4)		2)		1)		1)	
**Symptoms of anxiety (GAD-2)** [7]			**p<0.001**		**p<0.001**		**p=0.001**		**p=0.002**					**p<0.001**		**p<0.001**		**p<0.001**		**p<0.001**		
No	4.10 %	[3.0%,5.6%]	1.00		1.00		1.00		1.00		1160, 1011	9.60 %	[7.8%,11.7%]	1.00		1.00		1.00		1.00		996, 1103
Yes	10.60 %	[8.5%,13.3%]	2.78	(1.84-4.18)	2.36	(1.54-3.61)	2.34	(1.39-3.92)	2.36	(1.36-4.07)	820, 656	28.80 %	[24.4%, 33.6%]	3.82	(2.77-5.26)	3.26	(2.33-4.54)	2.90	(1.93-4.36)	2.66	(1.74-4.07)	495, 564
**Formed new relationship, past year**[8] **, ***			**p<0.001**		**p<0.001**		**p<0.001**		**p<0.001**					**p<0.001**		**p<0.001**		**p<0.001**		**p<0.001**		
No	3.2%	[2.4%,4.3%]	1.00		1.00		1.00		1.00		1640, 1390	7.4%	[5.8%,9.3%]	1.00		1.00		1.00		1.00		1153, 1289
Yes	25.5 %	[20.7%, 30.9%]	10.42	(6.92-15.69)	8.34	(5.33-13.06)	7.18	(4.22-12.22)	6.38	(3.24-12.59)	348, 285	45.8 %	[40.0%, 51.9%]	10.65	(7.53-15.06)	7.87	(5.47-11.32)	7.64	(4.99-11.71)	5.85	(3.55-9.66)	346, 385
**Total sexual partners, past year** [5] ***			**p<0.001**		**p<0.001**		**p=0.3315**		**p=0.54**					**p<0.001**		**p<0.001**		**p=0.36**		**p=0.54**		
0 or 1 partner	5.2%	[4.1%,6.4%]	1.00		1.00		1.00		1.00		1711, 1439	11.2 %	[9.4%,13.4%]	1.00		1.00		1.00		1.00		1192, 1346
2+ partners	22.5 %	[16.0%, 30.6%]	5.34	(3.31-8.62)	4.08	(2.49-6.67)	1.45	(0.69-3.05)	1.26	(0.60-2.67)	177, 144	37.7 %	[30.7%, 45.3%]	4.78	(3.29-6.95)	3.83	(2.58-5.69)	1.28	(0.76-2.16)	1.17	(0.71-1.95)	222, 237

**Previous same-sex experience, past 5 years** 6 ***			**p<0.001**		**p<0.001**		**p<0.001**		**p<0.001**					**p<0.001**		**p<0.001**		**p<0.001**		**p<0.001**		
No	5.8%	[4.7%,7.1%]	1.00		1.00		1.00		1.00		1837, 1606	14.1%	[12.1%, 16.2%]	1.00		1.00		1.00		1.00		1308, 1553
Yes	29.8%	[20.5%, 41.2%]	6.88	(3.99-11.84)	6.70	(3.89-11.52)	5.41	(2.75-10.64)	5.00	(2.48-10.08)	143, 65	36.8%	[27.8%, 46.8%]	3.56	(2.28-5.56)	3.37	(2.11-5.38)	2.93	(1.72-4.99)	2.85	(1.68-4.86)	183, 114
-	-	-	-	-	-	-	-	-	-	-	-	-										-
**Used an STI-related service, past year**			**p<0.001**		**p<0.001**		**p=0.45**		**p=0.60**					**p<0.001**		**p<0.001**		**p<0.001**		**p<0.001**		
No	5.80%	[4.7%,7.1%]	1.00		1.00		1.00		1.00		1788, 1504	12.80%	[11.0%, 15.0%]	1.00		1.00		1.00		1.00		1331, 1492
Yes	19.90%	[13.8%, 27.9%]	4.05	(2.48-6.63)	2.75	(1.61-4.70)	1.32	(0.64-2.73)	1.23	(0.57-2.65)	162, 135	53.80%	[44.3%, 63.1%]	7.91	(5.19-12.06)	6.40	(4.09-10.02)	4.03	(2.30-7.06)	3.83	(2.18-6.71)	141, 141

CI=confidence intervals. OR=odds ratio. aOR=age-adjusted odds ratio. AOR=adjusted odds ratio. PHQ-2=Patient Health Questionnaire (2 item). GAD-2=Generalized anxiety disorder (2 item)

* Age adjusted

** Sociodemographic adjusted (age, region, rurality, ethnicity, relationship formation)

*** Sociodemographic and behaviour adjusted (age, region, rurality, ethnicity, relationship formation, total partners in the past year, previous same-sex experience in the past 5 years)

† Men or women aged 18-44 who were sexually-experienced. Trans men and trans women are included in data for men and women, respectively. 31 women and 35 men responded 'prefer not to say' to questions about condom access or questions used for routing. These individuals are excluded from the denominator.

**** Unweighted denominator <30. Results not shown due to small denominator

[1] White includes all those who identify as White English, Welsh, Scottish, Northern Irish, British, Irish, Gypsy or Irish Traveller, or from any other White background.
[2] Mixed ethnicity includes those who identify as White and Black African, White and Black Caribbean, White and Asian or any other mixed or multiple ethnic background.
[3] Asian includes those who identify as Indian, Pakistani, Bangladeshi, Chinese or from any other Asian background
[4] Black includes those who identify as African, Caribbean, or from any other Black background.
[5] Includes both opposite-sex and same-sex partners
[6] Same-sex experience defined as oral/anal/vaginal sex
[7] Participants were classified as having symptoms of depression or anxiety if they scored three or more on the patient health questionnaire two item (PHQ-2) or generalised anxiety disorder two item (GAD-2) scales
[8] Formation of new romantic or sexual relationships in the past year instead of 'new sexual partners in the past year' because some new relationships may have delayed sex because of an unmet need for condoms

All percentages are weighted. These are row percentages which describe reported unmet need for condoms because of the pandemic within certain subgroups.

Supplementary table 3. Variations in reporting a chlamydia test among sexually-experienced women and men aged 18-44 years in the first year following the start of a national lockdown in Britain (23/03/2020) compared with Natsal-3 (2010-12)

	Women (sexually-experienced)														
	Natsal COVID (fieldwork 2021)							Natsal-3 (fieldwork 2010-12)							
	Weighted %	95% CI	OR	95% CI	aOR *	95% CI	Denominator † (unweighted, weighted)	Weighted %	95% CI	OR	95% CI	aOR *	95% CI	Denominator † (unweighted, weighted)	Interaction terms between surveys p-value
**All ages (18-44 years)**	7.3%	[6.2%,8.5%]	-	-	-	-	2145, 1824	25.1 %	[23.7%, 26.4%]	-	-	-	-	5004, 3546	

**Age (years)**			**p<0.001**							**p<0.001**					**p=0.06**
18-24	15.60%	[12.1%, 20.0%]	7.46	(4.18 - 13.31)	-	-	411, 328	54.5%	[51.5%, 57.5%]	11.97	(9.42 - 15.22)	-	-	1457, 833	
25-29	10.70%	[8.1%,13.8%]	4.79	(2.69 - 8.54)	-	-	545, 440	30.0%	[27.1%, 33.0%]	4.27	(3.33 - 5.49)	-	-	1357, 667	
30-34	4.90%	[3.2%,7.5%]	2.07	(1.06 - 4.05)	-	-	429, 355	16.5%	[14.2%, 19.2%]	1.98	(1.50 - 2.61)	-	-	1018, 645	
35-44	2.40%	[1.5%,3.9%]	1.00		-	-	760, 702	9.1%	[7.5%,11.0%]	1.00		-	-	1172, 1402	
**Region**			**p=0.11**		**p=0.11**					**p<0.001**		**p<0.001**			**p=0.71**
England/Wales	7.6%	[6.4%,9.0%]	1.00		1.00		1955, 1649	25.7%	[24.3%, 27.2%]	1.00		1.00		4578, 3233	
Scotland	4.1%	[1.9%,8.6%]	0.52	(0.23 - 1.17)	0.50	(0.22 - 1.16)	190, 175	17.9%	[14.8%, 21.5%]	0.63	(0.49 - 0.80)	0.56	(0.42 - 0.74)	426, 313	
**Rurality**			**p=0.25**		**p=0.30**			-	-	-	-	-	-	-	-
Urban	7.1%	[5.8%,8.7%]	1.00		1.00		1508, 1281	-	-	-	-	-	-	-	-
Rural	5.1%	[3.0%,8.6%]	0.71	(0.39 - 1.28)	0.71	(0.38 - 1.36)	254, 206	-	-	-	-	-	-	-	-
**Ethnicity**			**p<0.001**		**p=0.001**					**p<0.001**		**p<0.001**			**p=0.25**
White [1]	7.00%	[5.9%,8.3%]	1.00		1.00		1839, 1498	25.5%	[24.1%, 26.9%]	1.00		1.00		4362, 3052	
Mixed, multiple, or other [2]	16.60%	[8.4%,30.2%]	2.65	(1.19 - 5.90)	2.42	(1.06 - 5.55)	82, 62	34.8%	[26.6%, 43.9%]	1.56	(1.05 - 2.31)	1.19	(0.78 - 1.83)	180, 116	
Asian or Asian British [3]	1.80%	[0.5%,5.8%]	0.24	(0.07 - 0.83)	0.19	(0.05 - 0.71)	141, 159	11.6%	[8.1%,16.3%]	0.38	(0.26 - 0.57)	0.37	(0.24 - 0.58)	284, 237	
Black or Black British [4]	19.30%	[10.9%, 31.9%]	3.19	(1.59 - 6.40)	2.25	(1.12 - 4.53)	63, 77	31.4%	[23.1%, 41.0%]	1.34	(0.87 - 2.05)	1.42	(0.91 - 2.19)	169, 134	

**Sexual identity**			**p=0.34**		**p=0.51**					**p=0.05**		**p=0.95**			**p=0.61**
Heterosexual/straight	7.0%	[5.9%,8.3%]	1.00		1.00		1845, 1728	24.9%	[23.5%, 26.3%]	1.00		1.00		4786, 3400	
Gay or lesbian	16.6%	[8.4%,30.2%]	0.61	(0.18 - 2.06)	0.46	(0.12 - 1.79)	66, 20	20.6%	[11.7%, 33.5%]	0.78	(0.40 - 1.53)	0.83	(0.37 - 1.86)	64, 47	
Bisexual	1.8%	[0.5%,5.8%]	1.43	(0.85 - 2.40)	1.07	(0.62 - 1.84)	187, 44	35.9%	[27.8%, 45.0%]	1.70	(1.15 - 2.49)	1.09	(0.67 - 1.79)	131, 83	
Other	-	-	-	-	-	-	28, 11 **	-	-	-	-	-	-	14, 10 **	
**Social grade**			**p=0.16**		**p=0.51**			-	-	-	-	-	-	-	-
AB Higher and intermediate managerial/administrative/professional occupation	7.20%	[5.1%,10.0%]	1.00		1.00		500, 393	-	-	-	-	-	-	-	-
C1 Supervisory, clerical and junior managerial/administrative/professional occupations/C2 Skilled manual occupations	6.30%	[4.9%,8.1%]	0.87	(0.56 - 1.36)	0.86	(0.55 - 1.36)	1042, 933	-	-	-	-	-	-	-	-
D Semi-skilled and unskilled manual occupations/E On state benefit, unemployed and lowest grade occupations	9.10%	[6.9%,11.9%]	1.29	(0.81 - 2.07)	1.10	(0.68 - 1.79)	603, 498	-	-	-	-	-	-	-	-
**Highest education qualification**			**p=0.52**		**p=0.97**					**p=0.001**		**p=0.93**			**p=0.53**
Degree	6.70%	[5.3%,8.5%]	1.00		1.00		1086, 930	21.4%	[19.1%, 23.8%]	1.00		1.00		1441, 1123	
Below degree	7.70%	[6.1%,9.7%]	1.16	(0.81 - 1.67)	0.96	(0.66 - 1.39)	955, 802	26.9%	[25.3%, 28.7%]	1.36	(1.15 - 1.60)	1.03	(0.86 - 1.23)	3239, 2221	
No qualifications	9.30%	[5.0%,16.7%]	1.33	(0.70 - 2.94)	0.95	(0.42 - 2.15)	104, 92	25.1%	[20.3%, 30.5%]	1.23	(0.91 - 1.67)	1.06	(0.76 - 1.47)	318, 199	
**Born outside the UK**			**p=0.56**		**p=0.54**			-	-	-	-	-	-	-	-

No	7.20%	[6.0%,8.5%]	1.00		1.00		1808, 1509	-	-	-	-	-	-	-	-
Yes	8.20%	[5.4%,12.1%]	1.15	(0.72 - 1.86)	1.16	(0.72 - 1.88)	321, 299	-	-	-	-	-	-	-	-
**Relationship status**			**p<0.001**		**p=0.001**					**p<0.001**		**p<0.001**			**p=0.40**
Married/steady and living together	4.70%	[3.6%,6.1%]	1.00		1.00		1277, 1104	15.7%	[14.4%, 17.2%]	1.00		1.00		2696, 2273	
Steady not living together	11.50%	[7.9%,16.4%]	2.63	(1.61 - 4.32)	1.91	(1.14 - 3.18)	255, 202	47.4%	[43.8%, 51.1%]	4.84	(4.04 - 5.80)	2.37	(1.94 - 2.89)	1016, 532	
Not in a steady relationship	11.10%	[8.6%,14.2%]	2.53	(1.71 - 3.75)	2.05	(1.37 - 3.08)	607, 512	37.4%	[34.3%, 40.7%]	3.21	(2.70 - 3.82)	2.09	(1.73 - 2.53)	1281, 735	
**Days drinking, past 7 days**			**p=0.05**		**p=0.04**			-	-	-	-	-	-	-	-
0 days	5.6%	[4.2%,7.3%]	1.00		1.00		933, 805	-	-	-	-	-	-	-	-
1-2 days	8.4%	[6.5%,10.8%]	1.56	(1.04 - 2.33)	1.36	(0.90 - 2.05)	808, 678	-	-	-	-	-	-	-	-
3-4 days	8.1%	[5.2%,12.6%]	1.50	(0.85 - 2.65)	1.27	(0.71 - 2.28)	265, 228	-	-	-	-	-	-	-	-
5-7 days	11.3%	[6.8%,18.3%]	2.16	(1.14 - 4.09)	2.50	(1.32 - 4.73)	133, 107	-	-	-	-	-	-	-	-
**Currently smoker**			**p=0.42**		**p=0.65**					**p<0.001**		**p<0.001**			**p=0.18**
No	7.0%	[5.7%,8.4%]	1.00		1.00		1647, 1403	22.1%	[20.6%, 23.7%]	1.00		1.00		3418, 2544	
Yes	8.1%	[5.8%,11.3%]	1.19	(0.78 - 1.79)	1.10	(0.72 - 1.70)	486, 411	32.5%	[29.9%, 35.2%]	1.69	(1.46 - 1.97)	1.49	(1.28 - 1.75)	1586, 1002	
**Importance of sexual health, past year**			**p<0.001**		**p<0.001**			-	-	-	-	-	-	-	-
Very important/somewhat important	9.8%	[8.3%,11.7%]	1.00		1.00		1372, 1166	-	-	-	-	-	-	-	-
Not very important/not important	2.3%	[1.4%,3.9%]	0.22	(0.12 - 0.39)	0.24	(0.14 - 0.43)	605, 517	-	-	-	-	-	-	-	-

This does not apply to me	3.6%	[1.2%,10.6%]	0.34	(0.11 - 1.10)	0.39	(0.12 - 1.24)	128, 108	-	-	-	-	-	-	-	-
**Symptoms of depression (PHQ-2) [5]**			**p=0.35**		**p=0.55**					**p=0.02**		**p=0.08**			**p=0.05**
No	6.7%	[5.4%,8.3%]	1.00		1.00		1300, 1119	24.6%	[23.2%, 26.0%]	1.00		1.00		4389, 3139	
Yes	7.9%	[6.1%,10.2%]	1.19	(0.83 - 1.71)	0.89	(0.60 - 1.31)	805, 671	29.4%	[25.7%, 33.4%]	1.28	(1.05 - 1.55)	1.22	(0.98 - 1.52)	605, 399	
**Symptoms of anxiety (GAD-2) [5]**			**p=0.10**		**p=0.69**			-	-	-	-	-	-	-	-
No	6.5%	[5.2%,8.1%]	1.00		1.00		1228, 1075	-	-	-	-	-	-	-	-
Yes	8.5%	[6.7%,10.8%]	1.34	(0.94 - 1.90)	1.08	(0.74 - 1.57)	899, 732	-	-	-	-	-	-	-	-
**Total sexual partners, past year [6]**			**p<0.001**		**p<0.001**					**p<0.001**		**p<0.001**			**p=0.31**
0 partners	3.3%	[1.9%,5.5%]	1.00		1.00		472, 412	12.4%	[8.4%,17.9%]	1.00		1.00		280, 184	
1 partner	6.4%	[5.2%,7.9%]	2.02	(1.12 - 3.63)	2.21	(1.19 - 4.10)	1374, 1157	19.7%	[18.3%, 21.1%]	1.73	(1.12 - 2.67)	1.41	(0.87 - 2.29)	3687, 2758	
2+ partners	27.9%	[21.0%, 36.0%]	11.44	(5.91 - 22.14)	9.42	(4.77 - 18.63)	179, 146	54.2%	[50.4%, 57.9%]	8.32	(5.19 - 13.34)	4.69	(2.78 - 7.91)	998, 577	
**New sexual partners, past year [6]**			**p<0.001**		**p<0.001**					**p<0.001**		**p<0.001**			**p=0.05**
0 partners	4.5%	[3.6%,5.7%]	1.00		1.00		1695, 1443	17.7%	[16.4%, 19.0%]	1.00		1.00		3582, 2707	
1 partner	18.4%	[13.5%, 24.7%]	4.74	(3.06 - 7.36)	3.70	(2.33 - 5.89)	233, 196	43.1%	[39.1%, 47.1%]	3.52	(2.93 - 4.24)	2.30	(1.90 - 2.80)	854, 509	
2+ partners	36.8%	[26.3%, 48.7%]	12.23	(7.10 - 21.07)	9.54	(5.48 - 16.59)	91, 71	61.0%	[55.6%, 66.0%]	7.28	(5.72 - 9.26)	4.22	(3.21 - 5.54)	526, 299	
**Condom-less sex with a new partner on first occasion, past year [6]**			**p<0.001**		**p<0.001**			-	-	-	-	-	-	-	-

None	5.40%	[4.4%,6.5%]	1.00		1.00		1845, 1555	-	-	-	-	-	-	-	-
At least one	26.10%	[19.8%, 33.5%]	6.23	(4.11 - 9.44)	5.05	(3.27 - 7.79)	191, 168	-	-	-	-	-	-	-	-
**Previous same-sex experience, past 5 years [7]**			**p=0.09**		**p=0.13**					**p<0.001**		**p=0.04**			**p=0.61**
No	7.2%	[6.0%,8.5%]	1.00		1.00		1977, 1740	24.4%	[23.0%, 25.8%]	1.00		1.00		4709, 3361	
Yes	12.4%	[6.8%,21.5%]	1.83	(0.92 - 3.65)	1.71	(0.86 - 3.42)	143, 65	36.9%	[30.9%, 43.3%]	1.81	(1.37 - 2.40)	1.39	(1.01 - 1.92)	294, 185	
**Used an STI-related service, past year**			**p<0.001**		**p<0.001**					**p<0.001**		**p<0.001**			**p=0.01**
No	3.1%	[2.3%,4.1%]	1.00		1.00		1912, 1621	19.5%	[18.3%, 20.8%]	1.00		1.00		4465, 3235	
Yes	54.9%	[46.5%, 62.9%]	38.11	(24.60 - 59.03)	30.51	(19.37 - 48.05)	174, 146	83.1%	[78.8%, 86.7%]	20.23	(15.17 - 26.98)	13.05	(9.57 - 17.81)	532, 306	
**Unmet need for condoms, past year**			**p=0.07**		**p=0.57**			-	-	-	-	-	-	-	-
No	7.30%	[6.2%,8.7%]	1.00		1.00		1852, 1559	-	-	-	-	-	-	-	-
Yes	12.50%	[7.2%,20.9%]	1.81	(0.96 - 3.43)	1.22	(0.62 - 2.41)	134, 116	-	-	-	-	-	-	-	-

	**Men (sexually-experienced)**														
	**Natsal COVID (fieldwork 2021)**							**Natsal-3 (fieldwork 2010-12)**							
	**Weighted %**	**95% CI**	**OR**	**95% CI**	**aOR ***	**95% CI**	**Denominator † (unweighted, weighted)**	**Weighted %**	**95% CI**	**OR**	**95% CI**	**aOR ***	**95% CI**	**Denominator † (unweighted, weighted)**	**Interaction terms between surveys p-value**

**All ages (18-44 years)**	4.10%	[3.2%,5.3%]	-	-	-	-	1666, 1870	15.10%	[13.9%,16.3%]	-	-	-	-	3361, 3534	
**Age (years)**			**p=0.001**							**p<0.001**					**p=0.0070**
18-24	7.00%	[4.4%,10.9%]	3.75	(1.90 - 7.42)	-	-	358, 399	33.50%	[30.5%,36.7%]	11.79	(8.07 - 17.21)	-	-	1134, 860	
25-29	5.00%	[3.0%,8.3%]	2.63	(1.28 - 5.40)	-	-	355, 370	19.40%	[16.7%,22.4%]	5.62	(3.79 - 8.33)	-	-	823, 649	
30-34	5.00%	[2.7%,9.1%]	2.62	(1.18 - 5.83)	-	-	244, 287	9.50%	[7.2%,12.3%]	2.44	(1.55 - 3.86)	-	-	621, 645	
35-44	2.00%	[1.2%,3.1%]	1.00		-	-	709, 815	4.10%	[2.9%,5.7%]	1.00		-	-	783, 1380	
**Region**			**p=0.25**		**p=0.24**					**p=0.04**		**p=0.0206**			**p=0.6067**
England/Wales	4.30%	[3.3%,5.6%]	1.00		1.00		1526, 1702	15.50%	[14.2%,16.9%]	1.00		1.00		3080, 3232	
Scotland	2.10%	[0.6%,6.9%]	0.47	(0.13 - 1.70)	0.46	(0.12 - 1.68)	140, 168	10.20%	[6.7%,15.1%]	0.62	(0.39 - 0.98)	0.58	(0.37 - 0.92)	281, 302	
**Rurality**			**p=0.22**		**p=0.23**			-	-	-	-	-	-	-	-
Urban	4.10%	[3.1%,5.5%]	1.00		1.00		1245, 1397	-	-	-	-	-	-	-	-
Rural	1.90%	[0.6%,6.1%]	0.46	(0.14 - 1.57)	0.47	(0.14 - 1.61)	134, 158	-	-	-	-	-	-	-	-
**Ethnicity**			**p<0.001**		**p=0.001**					**p<0.001**		**p<0.001**			**p=0.45**
White [1]	3.50%	[2.7%,4.7%]	1.00		1.00		1360, 1509	15.40%	[14.1%,16.8%]	1.00		1.00		2929, 2997	
Mixed, multiple, or other [2]	17.10%	[7.6%,34.2%]	5.63	(2.14 - 14.83)	4.66	(1.77 - 12.21)	61, 78	25.50%	[17.3%,35.8%]	1.88	(1.14 - 3.08)	1.55	(0.96 - 2.51)	112, 112	
Asian or Asian British [3]	1.40%	[0.4%,5.2%]	0.39	(0.10 - 1.55)	0.35	(0.08 - 1.43)	146, 181	4.80%	[2.8%,8.1%]	0.28	(0.16 - 0.49)	0.31	(0.17 - 0.55)	198, 286	

Black or Black British [4]	9.30%	[4.0%,20.2%]	2.79	(1.07 - 7.24)	2.26	(0.87 - 5.84)	65, 72	20.50%	[13.4%, 30.1%]	1.42	(0.84 - 2.39)	1.58	(0.85 - 2.92)	116, 133	
**Sexual identity**			**p<0.001**		**p<0.001**					**p<0.001**		**p<0.001**			**p=0.58**
Heterosexual/straight	3.70%	[2.7%,4.9%]	1.00		1.00		1431, 1768	14.50%	[13.3%, 15.8%]	1.00		1.00		3236, 3422	
Gay	17.90%	[11.6%, 26.6%]	5.73	(3.17 - 10.34)	5.74	(3.11 - 10.60)	121, 49	42.10%	[29.7%, 55.7%]	4.29	(2.46 - 7.48)	4.54	(2.24 - 9.18)	82, 69	
Bisexual	5.60%	[1.8%,16.4%]	1.57	(0.46 - 5.34)	1.02	(0.27 - 3.82)	75, 22	16.90%	[7.4%,34.1%]	1.20	(0.47 - 3.06)	0.90	(0.35 - 2.30)	32, 34 ***	
Other	-	-	-	-	-	-	21, 12 **	-	-	-	-	-	-	8, 5 **	
**Social grade**			**p=0.01**		**p=0.01**			-	-	-	-	-	-	-	-
AB Higher and intermediate managerial/administrative/professional occupation	6.40%	[4.4%,9.1%]	1.00		1,00		580, 433	-	-	-	-	-	-	-	-
C1 Supervisory, clerical and junior managerial/administrative/professional occupations/C2 Skilled manual occupations	2.60%	[1.6%,4.1%]	0.39	(0.21 - 0.72)	0.39	(0.21 - 0.73)	691, 1010	-	-	-	-	-	-	-	-
D Semi-skilled and unskilled manual occupations/E On state benefit, unemployed and lowest grade occupations	5.40%	[3.3%,8.9%]	0.84	(0.44 - 1.63)	0.75	(0.39 - 1.43)	395, 427	-	-	-	-	-	-	-	-
**Highest education qualification**			**p<0.001**		**p=0.005**					**p<0.001**		**p=0.70**			**p=0.04**
Degree	3.10%	[2.1%,4.5%]	1.00		1.00		810, 845	11.40%	[9.5%,13.7%]	1.00		1.00		906, 1057	
Below degree	3.80%	[2.6%,5.5%]	1.23	(0.72 - 2.13)	1.10	(0.64 - 1.89)	750, 905	17.10%	[15.5%, 18.8%]	1.60	(1.26 - 2.02)	1.11	(0.87 - 1.42)	2277, 2273	
No qualifications	13.70%	[6.9%,25.2%]	4.97	(2.13 - 11.59)	3.93	(1.70 - 9.11)	106, 120	11.30%	[7.1%,17.4%]	0.98	(0.57 - 1.70)	1.03	(0.55 - 1.92)	173, 200	

**Born outside the UK**			**p=0.38**		**p=0.47**			-	-	-	-	-	-	-	-
No	4.30%	[3.3%,5.6%]	1.00		1.00		1491, 1681	-	-	-	-	-	-	-	-
Yes	2.70%	[1.0%,7.3%]	0.62	(0.21 - 1.81)	0.67	(0.23 - 1.97)	160, 174	-	-	-	-	-	-	-	-
**Relationship status**			**p=0.54**		**p=0.45**					**p<0.001**		**p<0.001**			**p<0.001**
Married/steady and living together	4.00%	[2.8%,5.7%]	1.00		1.00		972, 1099	7.00%	[5.8%,8.3%]	1.00		1.00		1622, 2153	
Steady not living together	2.80%	[1.0%,7.6%]	0.70	(0.23 - 2.10)	0.47	(0.14 - 1.55)	153, 174	30.40%	[26.7%, 34.5%]	5.84	(4.46 - 7.65)	2.58	(1.87 - 3.56)	676, 531	
Not in a steady relationship	4.90%	[3.3%,7.0%]	1.23	(0.71 - 2.12)	0.97	(0.54 - 1.74)	526, 577	25.90%	[23.1%, 29.0%]	4.68	(3.64 - 6.01)	2.30	(1.72 - 3.07)	1055, 842	
**Days drinking, past 7 days**			**p=0.03**		**p=0.03**			-	-	-	-	-	-	-	-
0 days	3.90%	[2.3%,6.6%]	1.00		1.00		505, 559	-	-	-	-	-	-	-	-
1-2 days	3.30%	[2.1%,5.1%]	0.83	(0.40 - 1.71)	0.79	(0.38 - 1.63)	667, 780	-	-	-	-	-	-	-	-
3-4 days	7.20%	[4.8%,10.8%]	1.92	(0.95 - 3.89)	1.91	(0.94 - 3.89)	326, 350	-	-	-	-	-	-	-	-
5-7 days	2.50%	[1.0%,6.2%]	0.63	(0.21 - 1.89)	0.64	(0.21 - 1.92)	162, 174	-	-	-	-	-	-	-	-
**Currently smoker**			**p<0.001**		**p<0.001**					**p<0.001**		**p<0.001**			**p=0.04**
No	2.30%	[1.6%,3.2%]	1.00		1.00		1135, 1255	12.40%	[11.1%, 13.9%]	1.00		1.00		2181, 2388	
Yes	8.00%	[5.7%,11.3%]	3.77	(2.24 - 6.34)	3.39	(1.99 - 5.78)	516, 599	20.60%	[18.1%, 23.3%]	1.83	(1.49 - 2.25)	1.64	(1.32 - 2.02)	1180, 1146	
**Importance of sexual health, past year**			**p=0.002**		**p=0.002**			-	-	-	-	-	-	-	-

Very important/somewhat important	5.40%	[4.1%,7.2%]	1.00		1.00		1037, 1184	-	-	-	-	-	-	-	-
Not very important/not important	2.10%	[1.2%,3.7%]	0.37	(0.19 - 0.72)	0.36	(0.19 - 0.70)	494, 541	-	-	-	-	-	-	-	-
This does not apply to me	0.60%	[0.1%,4.5%]	0.11	(0.02 - 0.84)	0.14	(0.02 - 1.05)	106, 111	-	-	-	-	-	-	-	-
**Symptoms of depression (PHQ-2)** [5]			**p=0.06**		**p=0.22**					**p=0.06**		**p=0.17**			**p=0.82**
No	3.20%	[2.2%,4.5%]	1.00		1.00		1004, 1127	14.70%	[13.4%,16.0%]	1.00		1.00		3012, 3196	
Yes	5.20%	[3.5%,7.7%]	1.69	(0.97 - 2.94)	1.44	(0.81 - 2.56)	626, 706	18.90%	[14.8%,23.8%]	1.35	(0.98 - 1.85)	1.28	(0.90 - 1.82)	343, 332	
**Symptoms of anxiety (GAD-2)** [5]			**p=0.004**		**p=0.02**			-	-	-	-	-	-	-	-
No	2.90%	[2.0%,4.1%]	1.00		1.00		1066, 1193	-	-	-	-	-	-	-	-
Yes	6.20%	[4.3%,8.9%]	2.23	(1.30 - 3.84)	1.94	(1.11 - 3.38)	570, 646	-	-	-	-	-	-	-	-
**Total sexual partners, past year** [6]			**p<0.001**		**p<0.001**					**p<0.001**		**p<0.001**			**p=0.004**
0 partners	2.60%	[1.5%,4.5%]	1.00		1.00		412, 462	1.20%	[0.4%,3.8%]	1.00		1.00		180, 168	
1 partner	2.00%	[1.3%,3.3%]	0.78	(0.36 - 1.65)	0.88	(0.41 - 1.90)	923, 1056	9.80%	[8.6%,11.1%]	9.08	(2.71 - 30.44)	11.24	(3.34 - 37.83)	2207, 2545	
2+ partners	15.60%	[10.8%,22.1%]	6.93	(3.38 - 14.18)	6.60	(3.22 - 13.55)	223, 238	34.60%	[31.1%,38.3%]	44.23	(13.12 - 149.21)	35.10	(10.38 - 118.69)	945, 795	
**New sexual partners, past year** [6]			**p<0.001**		**p<0.001**					**p<0.001**		**p<0.001**			**p=0.18**
0 partners	1.70%	[1.1%,2.7%]	1.00		1.00		1180, 1336	8.20%	[7.1%,9.5%]	1.00		1.00		2059, 2462	
1 partner	7.40%	[4.6%,11.6%]	4.54	(2.31 - 8.92)	4.09	(2.06 - 8.09)	226, 257	24.90%	[21.5%,28.7%]	3.72	(2.86 - 4.82)	2.13	(1.62 - 2.80)	695, 576	
2+ partners	18.20%	[11.7%,27.2%]	12.65	(6.34 - 25.23)	10.70	(5.31 - 21.59)	146, 155	39.00%	[34.3%,43.9%]	7.16	(5.51 - 9.29)	4.31	(3.23 - 5.77)	573, 464	

**Condom-less sex with a new partner on first occasion, past year [6]**			**p<0.001**		**p<0.001**			-	-	-	-	-	-	-	-
None	2.20%	[1.5%,3.1%]	1.00		1.00		1288, 1446	-	-	-	-	-	-	-	-
At least one	13.20%	[9.1%,18.6%]	6.89	(3.91 - 12.15)	6.14	(3.46 - 10.87)	260, 297	-	-	-	-	-	-	-	-
**Previous same-sex experience, past 5 years [7]**			**p<0.001**		**p<0.001**					**p<0.001**		**p<0.001**			**p=0.04**
No	3.00%	[2.2%,4.2%]	1.00		1.00		1452, 1723	14.40%	[13.2%, 15.7%]	1.00		1.00		3231, 3422	
Yes	17.30%	[11.5%, 25.3%]	6.79	(3.77 - 12.24)	6.43	(3.51 - 11.78)	183, 115	35.10%	[26.2%, 45.1%]	3.21	(2.09 - 4.94)	3.01	(1.76 - 5.14)	130, 112	
**Used an STI-related service, past year**			**p<0.001**		**p<0.001**					**p<0.001**		**p<0.001**			**p<0.001**
No	2.40%	[1.6%,3.5%]	1.00		1.00		1457, 1648	9.90%	[8.9%,11.0%]	1.00		1.00		3044, 3283	
Yes	23.10%	[16.9%, 30.7%]	12.43	(7.06 - 21.88)	10.81	(5.78 - 20.23)	163, 163	84.0%	[79.1%, 87.9%]	47.70	(33.62 - 67.69)	36.84	(23.95 - 56.67)	314, 247	
**Unmet need for condoms, past year**			**p<0.001**		**p<0.001**			-	-	-	-	-	-	-	-
No	2.60%	[1.8%,3.6%]	1.00		1.00		1262, 1406	-	-	-	-	-	-	-	-
Yes	14.20%	[9.8%,20.1%]	6.30	(3.61 - 11.00)	5.23	(2.88 - 9.51)	238, 270	-	-	-	-	-	-	-	-

CI=confidence intervals. OR=odds ratio. aOR=age-adjusted odds ratio. PHQ-2=Patient Health Questionnaire (2 item). GAD-2=Generalized anxiety disorder (2 item)

[*] Age adjusted

[†] Men or women aged 18-44 who were sexually-experienced. Trans men and trans women are included in data for men and women, respectively. 18 women and 24 men in Natsal-COVID responded 'prefer not to say' to questions about chlamydia testing. 120 women and 66 men in Natsal-3 did not answer the question. These individuals are excluded from the denominator.

** Unweighted denominator <30. Results not shown due to small denominator
***Unweighted denominator <50. Results should be interpreted with caution due to small denominator.
[1] White includes all those who identify as White English, Welsh, Scottish, Northern Irish, British, Irish, Gypsy or Irish Traveller, or from any other White background.
[2] Mixed ethnicity includes those who identify as White and Black African, White and Black Caribbean, White and Asian or any other mixed or multiple ethnic background.
[3] Asian includes those who identify as Indian, Pakistani, Bangladeshi, Chinese or from any other Asian background
[4] Black includes those who identify as African, Caribbean, or from any other Black background.
[5] Participants were classified as having symptoms of depression or anxiety if they scored three or more on the patient health questionnaire two item (PHQ-2) or generalised anxiety disorder two item (GAD-2) scales
[6] Includes both opposite-sex and same-sex partners
[7] Same-sex experience defined as oral/anal/vaginal sex

All percentages are weighted. These are row percentages which describe reported chlamydia testing in the past year within certain subgroups.

Supplementary table 4. Variations in reporting an HIV test among sexually-experienced women and men aged 18-44 years in the first year following the start of a national lockdown in Britain (23/03/2020) compared with Natsal-3 (2010-12)

	**Women (sexually-experienced)**														
	**Natsal COVID (fieldwork 2021)**							**Natsal-3 (fieldwork 2010-12)**							
	**Weighted %**	**95% CI**	**OR**	**95% CI**	**aOR***	**95% CI**	**Denominator † (unweighted, weighted)**	**Weighted %**	**95% CI**	**OR**	**95% CI**	**aOR***	**95% CI**	**Denominator † (unweighted, weighted)**	**Interaction terms between surveys p-value**
**All ages (18-44 years)**	8.6%	[7.4%,1	-	-	-	-	2148,	10.4	[9.5%,1	-	-	-	-	4701,	

		0.0%]					1827	%	1.4%]					3331	
**Age (years)**			**p<0.001**							**p<0.001**					**p=0.92**
18-24	12.2%	[9.0%,16.4%]	2.85	(1.71 - 4.75)	-	-	411, 327	14.9%	[12.8%, 17.3%]	3.11	(2.22 - 4.35)	-	-	1367, 783	
25-29	11.8%	[9.0%,15.3%]	2.76	(1.70 - 4.47)	-	-	545, 440	13.7%	[11.5%, 16.1%]	2.81	(1.99 - 3.96)	-	-	1269, 622	
30-34	9.1%	[6.6%,12.4%]	2.06	(1.23 - 3.43)	-	-	430, 356	12.3%	[10.3%, 14.6%]	2.48	(1.74 - 3.53)	-	-	970, 614	
35-44	4.6%	[3.2%,6.6%]	1.00		-	-	762, 703	5.3%	[4.1%,7.0%]	1.00		-	-	1095, 1312	
**Region**			**p=0.01**		**p=0.008**					**p=0.02**		**p=0.02**			**p=0.29**
England/Wales	9.1%	[7.8%,10.7%]	1.00		1.00		1957, 1650	10.8%	[9.8%,11.8%]	1.00		1.00		4307, 3036	
Scotland	3.7%	[1.8%,7.2%]	0.38	(0.18 - 0.79)	0.37	(0.18 - 0.77)	191, 176	6.7%	[4.6%,9.7%]	0.59	(0.39 - 0.90)	0.59	(0.38 - 0.90)	394, 294	
**Rurality**			**p=0.10**		**p=0.13**			-	-	-	-	-	-	-	-
Urban	8.4%	[7.0%,10.2%]	1.00		1.00		1511, 1283	-	-	-	-	-	-	-	-
Rural	5.3%	[3.1%,8.9%]	0.61	(0.34 - 1.10)	0.62	(0.34 - 1.14)	255, 207	-	-	-	-	-	-	-	-
**Ethnicity**			**p<0.001**		**p<0.001**					**p<0.001**		**p<0.001**			**p=0.11**
White [1]	7.4%	[6.2%,8.7%]	1.00		1.00		1840, 1498	9.9%	[9.0%,10.9%]	1.00		1.00		4076, 2850	
Mixed, multiple, or other [2]	26.4%	[15.1%, 41.8%]	4.50	(2.19 - 9.25)	4.28	(2.01 - 9.12)	83, 63	15.0%	[10.0%, 22.1%]	1.61	(0.99 - 2.63)	1.39	(0.84 - 2.33)	173, 111	
Asian or Asian British [3]	5.5%	[2.8%,10.9%]	0.74	(0.35 - 1.57)	0.68	(0.31 - 1.48)	142, 159	7.2%	[4.4%,11.8%]	0.71	(0.41 - 1.23)	0.74	(0.42 - 1.29)	277, 235	
Black or Black British [4]	23.6%	[14.2%, 36.6%]	3.88	(2.02 - 7.46)	3.15	(1.64 - 6.02)	63, 77	23.1%	[16.2%, 32.0%]	2.74	(1.73 - 4.33)	2.80	(1.80 - 4.33)	166, 128	

**Sexual identity**			**p=0.65**		**p=0.39**					**p=0.44**		**p=0.79**			**p=0.21**
Heterosexual/straight	8.8%	[7.5%,10.3%]	1.00		1.00		1848, 1730	10.3%	[9.3%,11.3%]	1.00		1.00		4494, 3191	
Gay or lesbian	5.5%	[1.9%,14.6%]	0.60	(0.20 - 1.80)	0.53	(0.17 - 1.65)	65, 19	12.3%	[6.0%,23.4%]	1.23	(0.56 - 2.70)	1.29	(0.59 - 2.80)	60, 45	
Bisexual	8.2%	[4.9%,13.4%]	0.93	(0.52 - 1.64)	0.77	(0.43 - 1.37)	187, 44	14.8%	[9.4%,22.5%]	1.52	(0.90 - 2.56)	1.24	(0.72 - 2.14)	125, 80	
Other	-		-	-	-	-	29, 12**	-		-	-	-	-	14, 10**	
**Social grade**			**p=0.27**		**p=0.48**			-	-	-	-	-	-	-	-
AB Higher and intermediate managerial/administrative/professional occupation	9.4%	[7.0%,12.5%]	1.00		1.00		500, 393	-	-	-	-	-	-	-	-
C1 Supervisory, clerical and junior managerial/administrative/professional occupations/C2 Skilled manual occupations	7.5%	[5.9%,9.6%]	0.79	(0.52 - 1.20)	0.80	(0.53 - 1.22)	1044, 934	-	-	-	-	-	-	-	-
D Semi-skilled and unskilled manual occupations/E On state benefit, unemployed and lowest grade occupations	10.0%	[7.6%,12.9%]	1.07	(0.69 - 1.66)	0.98	(0.62 - 1.52)	604, 499	-	-	-	-	-	-	-	-
**Highest education qualification**			**p=0.29**		**p=0.09**					**p=0.58**		**p=0.09**			**p=0.67**
Degree	9.60%	[7.8%,11.8%]	1.00		1.00		1088, 931	11.1%	[9.5%,13.0%]	1.00		1.00		1372, 1066	
Below degree	7.40%	[5.8%,9.4%]	0.76	(0.53 - 1.07)	0.68	(0.48 - 0.96)	955, 804	10.0%	[8.9%,11.2%]	0.89	(0.72 - 1.11)	0.78	(0.63 - 0.98)	3021, 2074	
No qualification	8.80%	[4.5%,16.3%]	0.91	(0.43 - 1.91)	0.70	(0.32 - 1.55)	105, 92	10.5%	[7.3%,14.8%]	0.94	(0.61 - 1.45)	0.88	(0.57 - 1.36)	302, 186	
**Born outside the UK**			**p=0.13**		**p=0.14**			-	-	-	-	-	-	-	-

No	8.1%	[6.9%,9.6%]	1.00		1.00		1908, 1509	-	-	-	-	-	-	-	-
Yes	11.1%	[7.7%,15.9%]	1.41	(0.90 - 2.22)	1.41	(0.90 - 2.22)	323, 301	-	-	-	-	-	-	-	-
**Relationship status**			**p=0.003**		**p=0.02**					**p=0.003**		**p=0.69**			**p=0.17**
Married/steady and living together	6.80%	[5.5%,8.5%]	1.00		1.00		1277, 1105	9.2%	[8.1%,10.5%]	1.00		1.00		2518, 2132	
Steady not living together	8.20%	[5.3%,12.6%]	1.22	(0.72 - 2.07)	1.01	(0.58 - 1.73)	254, 201	12.8%	[10.5%, 15.5%]	1.44	(1.11 - 1.88)	0.93	(0.70 - 1.24)	951, 498	
Not in a steady relationship	12.30%	[9.5%,15.8%]	1.91	(1.32 - 2.76)	1.69	(1.15 - 2.48)	611, 515	12.5%	[10.5%, 14.9%]	1.41	(1.11 - 1.79)	1.06	(0.83 - 1.35)	1206, 687	
**Days drinking, past 7 days**			**p=0.12**		**p=0.13**			-	-	-	-	-	-	-	-
0 days	7.1%	[5.4%,9.1%]	1.00		1.00		935, 807	-	-	-	-	-	-	-	-
1-2 days	9.8%	[7.7%,12.4%]	1.43	(0.97 - 2.10)	1.31	(0.89 - 1.93)	810, 680	-	-	-	-	-	-	-	-
3-4 days	8.8%	[5.7%,13.3%]	1.26	(0.73 - 2.18)	1.14	(0.67 - 1.95)	265, 227	-	-	-	-	-	-	-	-
5-7 days	12.7%	[7.8%,20.1%]	1.93	(1.05 - 3.54)	2.04	(1.10 - 3.78)	132, 107	-	-	-	-	-	-	-	-
**Currently smoker**			**p=0.03**		**p=0.05**					**p=0.96**		**p=0.46**			**p=0.04**
No	7.7%	[6.5%,9.3%]	1.00		1.00		1652, 1408	10.4%	[9.3%,11.6%]	1.00		1.00		3235, 2407	
Yes	11.5%	[8.5%,15.4%]	1.55	(1.05 - 2.28)	1.49	(1.00 - 2.21)	483, 408	10.5%	[8.9%,12.3%]	1.01	(0.80 - 1.26)	0.92	(0.73 - 1.15)	1466, 923	
**Importance of sexual health, past year**			**p<0.001**		**p<0.001**			-	-	-	-	-	-	-	-
Very important/somewhat important	10.9%	[9.1%,12.8%]	1.00		1.00		1370, 1164	-	-	-	-	-	-	-	-
Not very important/not important	4.5%	[3.1%,6.7%]	0.39	(0.25 - 0.61)	0.42	(0.27 - 0.67)	606, 517	-	-	-	-	-	-	-	-

This does not apply to me	4.7%	[1.8%,11.8%]	0.40	(0.15 - 1.12)	0.43	(0.16 - 1.20)	130, 109	-	-	-	-	-	-	-	-
**Symptoms of depression (PHQ-2) [5]**			**p=0.55**		**p=0.75**					**p=0.81**		**p=0.89**			**p=0.71**
No	8.1%	[6.6%,9.9%]	1.00		1.00		1305, 1124	10.3%	[9.4%,11.4%]	1.00		1.00		4132, 2945	
Yes	9.0%	[6.9%,11.5%]	1.11	(0.78 - 1.59)	0.94	(0.66 - 1.35)	803, 668	10.7%	[8.2%,13.9%]	1.04	(0.76 - 1.42)	1.02	(0.75 - 1.40)	559, 376	
**Symptoms of anxiety (GAD-2) [5]**			**p=0.24**		**p=0.68**			-	-	-	-	-	-	-	-
No	7.9%	[6.4%,9.8%]	1.00		1.00		1231, 1077	-	-	-	-	-	-	-	-
Yes	9.5%	[7.6%,12.0%]	1.23	(0.87 - 1.72)	1.08	(0.76 - 1.52)	899, 731	-	-	-	-	-	-	-	-
**Total sexual partners, past year [6]**			**p<0.001**		**p<0.001**					**p<0.001**		**p<0.001**			**p<0.001**
0 partners	7.6%	[5.1%,11.2%]	1.00		1.00		473, 413	4.6%	[2.3%,8.7%]	1.00		1.00		266, 175	
1 partner	6.6%	[5.4%,8.1%]	0.86	(0.53 - 1.38)	0.87	(0.54 - 1.42)	1376, 1159	9.2%	[8.2%,10.2%]	2.11	(1.04 - 4.26)	1.92	(0.95 - 3.89)	3447, 2573	
2+ partners	32.0%	[24.6%,40.4%]	5.73	(3.27 - 10.05)	5.03	(2.87 - 8.80)	179, 146	19.1%	[16.3%,22.3%]	4.95	(2.36 - 10.37)	3.57	(1.71 - 7.46)	926, 533	
**New sexual partners, past year [6]**			**p<0.001**		**p<0.001**					**p<0.001**		**p<0.001**			**p=0.001**
0 partners	6.6%	[5.4%,8.1%]	1.00		1.00		1698, 1446	8.5%	[7.6%,9.6%]	1.00		1.00		3348, 2521	
1 partner	16.5%	[11.9%,22.3%]	2.79	(1.81 - 4.30)	2.43	(1.57 - 3.74)	233, 195	13.8%	[11.3%,16.8%]	1.72	(1.31 - 2.26)	1.38	(1.05 - 1.83)	794, 475	
2+ partners	37.5%	[26.7%,49.6%]	8.48	(4.94 - 14.57)	7.29	(4.22 - 12.61)	91, 71	22.9%	[18.7%,27.6%]	3.18	(2.39 - 4.23)	2.36	(1.75 - 3.18)	494, 282	
**Condom-less sex with a new partner on first occasion, past year [6]**			**p<0.001**		**p<0.001**			-	-	-	-	-	-	-	-

None	7.3%	[6.1%,8.8%]	1.00		1.00		1848, 1558	-	-	-	-	-	-	-	-
At least one	23.8%	[17.8%, 31.1%]	3.96	(2.61 - 6.01)	3.44	(2.26 - 5.23)	190, 167	-	-	-	-	-	-	-	-
**Previous same-sex experience, past 5 years** [7]			**p=0.005**		**p=0.009**					**p=0.008**		**p=0.07**			**p=0.20**
No	8.3%	[7.0%,9.7%]	1.00		1.00		1979, 1742	10.1%	[9.2%,11.2%]	1.00		1.00		4428, 3154	
Yes	17.9%	[10.8%, 28.1%]	2.41	(1.31 - 4.45)	2.27	(1.23 - 4.19)	143, 65	15.5%	[11.5%, 20.5%]	1.63	(1.13 - 2.33)	1.41	(0.98 - 2.03)	271, 174	
**Pregnant in past year or currently pregnant**			**p<0.001**		**p<0.001**					**p<0.001**		**p<0.001**			**p=0.01**
No	6.9%	[5.6%,8.4%]	1.00		1.00		1658, 1393	6.4%	[5.6%,7.3%]	1.00		1.00		3878, 2815	
Yes	23.3%	[18.0%, 29.5%]	4.11	(2.78 - 6.06)	3.94	(2.65 - 5.86)	221, 191	33.8%	[30.2%, 37.7%]	7.50	(6.04 - 9.31)	7.06	(5.65 - 8.81)	798, 494	
**Used an STI-related service, past year**			**p<0.001**		**p<0.001**					**p<0.001**		**p<0.001**			**p=0.02**
No	4.9%	[3.9%,6.0%]	1.00		1.00		1917, 1625	6.9%	[6.1%,7.7%]	1.00		1.00		4151, 3003	
Yes	51.6%	[43.3%, 59.7%]	20.87	(13.95 - 31.23)	20.03	(13.03 - 30.78)	173, 145	47.4%	[42.5%, 52.3%]	12.21	(9.67 - 15.40)	10.76	(8.40 - 13.79)	512, 297	
**Unmet need for condoms, past year**			**p<0.001**		**p<0.001**			-	-	-	-	-	-	-	-
No	8.00%	[6.8%,9.5%]	1.00		1.00		1856, 1563	-	-	-	-	-	-	-	-
Yes	19.10%	[12.5%, 28.1%]	2.70	(1.58 - 4.62)	2.16	(1.22 - 3.85)	133, 115	-	-	-	-	-	-	-	-

	**Men (sexually-experienced)**		
	**Natsal COVID (fieldwork 2021)**	**Natsal-3 (fieldwork 2010-12)**	

	Weighted %	95% CI	OR	95% CI	aOR *	95% CI	Denominator † (unweighted, weighted)	Weighted %	95% CI	OR	95% CI	aOR *	95% CI	Denominator † (unweighted, weighted)	Interaction terms between surveys p-value
**All ages (18-44 years)**	6.5%	[5.4%,7.9%]	-	-	-	-	1668, 1868	6.00 %	[5.1%,7.0%]	-	-	-	-	3198, 3366	
**Age (years)**			**p=0.005**							**p=0.002**					**p=0.49**
18-24	8.6%	[5.9%,12.4%]	2.28	(1.31 - 3.97)	-	-	359, 395	6.40 %	[5.0%,8.2%]	1.71	(1.06 - 2.77)	-	-	1082, 820	
25-29	7.8%	[5.3%,11.4%]	2.05	(1.17 - 3.58)	-	-	357, 372	9.00 %	[7.1%,11.3%]	2.46	(1.54 - 3.93)	-	-	778, 614	
30-34	9.4%	[6.0%,14.3%]	2.51	(1.37 - 4.60)	-	-	243, 287	6.90 %	[5.0%,9.5%]	1.85	(1.10 - 3.11)	-	-	596, 617	
35-44	4.0%	[2.8%,5.6%]	1.00		-	-	709, 815	3.90 %	[2.6%,5.7%]	1.00		-	-	742, 1315	
**Region**			**p=0.14**		**p=0.14**					**p=0.13**		**p=0.13**			**p=0.67**
England/Wales	6.8%	[5.6%,8.3%]	1.00		1.00		1529, 1703	6.20 %	[5.3%,7.2%]	1.00		1.00		2933, 3082	
Scotland	3.4%	[1.4%,8.4%]	0.49	(0.19 - 1.28)	0.48	(0.18 - 1.27)	139, 165	4.00 %	[2.3%,6.9%]	0.63	(0.34 - 1.15)	0.63	(0.34 - 1.14)	265, 285	
**Rurality**			**p=0.03**		**p=0.03**			-	-	-	-	-	-	-	-
Urban	7.1%	[5.7%,8.8%]	1.00		1.00		1243, 1393	-	-	-	-	-	-	-	-
Rural	2.2%	[0.8%,6.0%]	0.29	(0.10 - 0.86)	0.3	(0.10 - 0.87)	132, 155	-	-	-	-	-	-	-	-

**Ethnicity**			**p<0.001**		**p<0.001**					**p<0.001**		**p=0.0002**			**p=0.64**
White [1]	5.9%	[4.7%,7.3%]	1.00		1.00		1359, 1507	5.50%	[4.7%,6.5%]	1.00		1.00		2785, 2844	
Mixed, multiple, or other [2]	23.2%	[12.0%, 40.3%]	4.86	(2.11 - 11.20)	4.39	(1.93 - 9.94)	59, 71	14.00%	[7.7%,24.0%]	2.78	(1.40 - 5.54)	2.65	(1.31 - 5.33)	109, 111	
Asian or Asian British [3]	2.7%	[1.3%,5.6%]	0.45	(0.20 - 0.99)	0.41	(0.19 - 0.91)	149, 184	3.30%	[1.6%,6.8%]	0.58	(0.27 - 1.27)	0.60	(0.28 - 1.32)	190, 279	
Black or Black British [4]	15.3%	[8.2%,26.7%]	2.90	(1.38 - 6.08)	2.50	(1.17 - 5.34)	65, 72	15.60%	[9.5%,24.5%]	3.17	(1.76 - 5.73)	3.23	(1.78 - 5.86)	108, 126	
**Sexual identity**			**p<0.001**		**p<0.001**					**p<0.001**		**p<0.001**			**p=0.37**
Heterosexual/straight	5.9%	[4.7%,7.4%]	1.00		1.00		1432, 1766	5.30%	[4.5%,6.3%]	1.00		1.00		3074, 3255	
Gay or lesbian	24.5%	[17.3%, 33.4%]	5.16	(3.14 - 8.47)	5.13	(3.09 - 8.53)	121, 49	36.00%	[24.3%, 49.5%]	9.97	(5.54 - 17.91)	9.68	(5.36 - 17.49)	82, 69	
Bisexual	9.5%	[4.3%,19.3%]	1.66	(0.70 - 3.95)	1.32	(0.54 - 3.20)	74, 20	7.10%	[2.1%,21.5%]	1.35	(0.37 - 4.91)	1.29	(0.36 - 4.60)	31, 33 ***	
Other	-	-	-	-	-	-	22, 12 **	-	-	-	-	-	-	8, 6 **	
**Social grade**			**p=0.01**		**p=0.01**			-	-	-	-	-	-	-	-
AB Higher and intermediate managerial/administrative/professional occupation	9.5%	[7.2%,12.5%]	1.00		1.00		583, 435	-	-	-	-	-	-	-	-
C1 Supervisory, clerical and junior managerial/administrative/professional occupations/C2 Skilled manual occupations	4.9%	[3.5%,6.9%]	0.50	(0.31 - 0.79)	0.50	(0.31 - 0.80)	691, 1006	-	-	-	-	-	-	-	-
D Semi-skilled and unskilled manual occupations/E On state benefit, unemployed and lowest grade occupations	7.3%	[4.9%,10.7%]	0.75	(0.44 - 1.26)	0.70	(0.41 - 1.17)	394, 426	-	-	-	-	-	-	-	-
**Highest education qualification**			**p=0.01**		**p=0.03**					**p=0.08**		**p=0.04**			**p=0.07**

Degree	6.40%	[4.8%,8.4%]	1.00		1.00		813, 846	7.50%	[5.8%,9.6%]	1.00		1.00		868, 1018	
Below degree	5.60%	[4.1%,7.6%]	0.87	(0.56 - 1.35)	0.80	(0.51 - 1.27)	750, 901	5.50%	[4.5%,6.6%]	0.71	(0.51 - 0.99)	0.66	(0.48 - 0.92)	2162, 2152	
No qualification	14.60%	[8.5%,24.1%]	2.52	(1.27 - 4.99)	2.12	(1.03 - 4.37)	106, 121	3.80%	[1.4%,9.5%]	0.48	(0.17 - 1.35)	0.50	(0.18 - 1.41)	163, 191	
**Born outside the UK**			**p=0.28**		**p=0.33**			-	-	-	-	-	-	-	-
No	6.8%	[5.5%,8.3%]	1.00		1.00		1492, 1678	-	-	-	-	-	-	-	-
Yes	4.6%	[2.2%,9.0%]	0.65	(0.31 - 1.40)	0.68	(0.32 - 1.47)	160, 174	-	-	-	-	-	-	-	-
**Relationship status**			**p=0.82**		**p=0.98**					**p<0.001**		**p=0.003**			**p=0.16**
Married/steady and living together	6.30%	[4.8%,8.2%]	1.00		1.00		975, 1098	4.30%	[3.3%,5.5%]	1.00		1.00		1535, 2045	
Steady not living together	7.60%	[4.2%,13.2%]	1.22	(0.62 - 2.41)	0.97	(0.46 - 2.05)	153, 174	9.00%	[6.8%,11.8%]	2.22	(1.52 - 3.24)	2.06	(1.29 - 3.29)	640, 504	
Not in a steady relationship	6.90%	[4.9%,9.5%]	1.10	(0.70 - 1.73)	0.95	(0.59 - 1.54)	524, 574	8.10%	[6.4%,10.2%]	1.98	(1.38 - 2.84)	1.86	(1.24 - 2.79)	1006, 802	
**Days drinking, past 7 days**			**p=0.002**		**p=0.001**			-	-	-	-	-	-	-	-
0 days	5.4%	[3.6%,7.9%]	1.00		1.00		509, 563	-	-	-	-	-	-	-	-
1-2 days	5.1%	[3.6%,7.1%]	0.94	(0.54 - 1.63)	0.91	(0.52 - 1.58)	667, 777	-	-	-	-	-	-	-	-
3-4 days	12.0%	[8.6%,16.6%]	2.42	(1.38 - 4.22)	2.41	(1.38 - 4.23)	324, 348	-	-	-	-	-	-	-	-
5-7 days	6.0%	[3.2%,10.8%]	1.12	(0.52 - 2.41)	1.15	(0.54 - 2.48)	161, 171	-	-	-	-	-	-	-	-
**Currently smoker**			**p<0.001**		**p<0.001**					**p=0.03**		**p=0.05**			**p<0.001**
No	3.30%	[2.5%,4.4%]	1.00		1.00		1132, 1253	5.30%	[4.4%,6.5%]	1.00		1.00		2086, 2290	

Yes	12.90%	[10.0%, 16.5%]	4.33	(2.86 - 6.57)	4.10	(2.69 - 6.26)	520, 600	7.40%	[5.9%,9.1%]	1.41	(1.04 - 1.91)	1.36	(1.00 - 1.83)	1112, 1076	
**Importance of sexual health, past year**			**p=0.002**		**p=0.002**			-	-	-	-	-	-	-	-
Very important/somewhat important	8.2%	[6.6%,10.2%]	1.00		1.00		1035, 1178	-	-	-	-	-	-	-	-
Not very important/not important	3.4%	[2.1%,5.4%]	0.39	(0.23 - 0.67)	0.38	(0.22 - 0.67)	495, 541	-	-	-	-	-	-	-	-
This does not apply to me	2.6%	[0.5%,11.2%]	0.29	(0.06 - 1.42)	0.33	(0.07 - 1.63)	108, 113	-	-	-	-	-	-	-	-
**Symptoms of depression (PHQ-2)** [5]			**p=0.001**		**p=0.005**					**p=0.27**		**p=0.28**			**p=0.25**
No	4.7%	[3.5%,6.3%]	1.00		1.00		1004, 1128	5.80%	[4.9%,6.9%]	1.00		1.00		2862, 3038	
Yes	9.1%	[6.9%,11.8%]	2.04	(1.33 - 3.14)	1.88	(1.21 - 2.93)	627, 702	7.50%	[5.0%,11.1%]	1.31	(0.82 - 2.08)	1.29	(0.81 - 2.05)	330, 323	
**Symptoms of anxiety (GAD-2)** [5]			**p=0.001**		**p=0.006**			-	-	-	-	-	-	-	-
No	5.0%	[3.8%,6.6%]	1.00		1.00		1065, 1192	-	-	-	-	-	-	-	-
Yes	9.5%	[7.2%,12.3%]	1.98	(1.30 - 3.02)	1.82	(1.19 - 2.79)	573, 645	-	-	-	-	-	-	-	-
**Total sexual partners, past year** [6]			**p<0.001**		**p<0.001**					**p<0.001**		**p<0.001**			**p=0.02**
0 partners	4.1%	[2.6%,6.6%]	1.00		1.00		413, 461	0.30%	[0.0%,2.4%]	1.00		1.00		169, 158	
1 partner	4.3%	[3.1%,6.0%]	1.05	(0.57 - 1.92)	1.12	(0.60 - 2.10)	921, 1054	4.20%	[3.4%,5.4%]	13.44	(1.81 - 99.93)	13.52	(1.82 - 100.59)	2086, 1404	
2+ partners	19.3%	[14.3%, 25.6%]	5.54	(3.00 - 10.22)	5.37	(2.90 - 9.92)	221, 233	12.30%	[10.0%, 15.0%]	42.45	(5.73 - 314.60)	41.25	(5.52 - 308.35)	902, 762	
**New sexual partners, past year** [6]			**p<0.001**		**p<0.001**					**p<0.001**		**p<0.001**			**p=0.43**

0 partners	3.5%	[2.5%,4.9%]	1.00		1.00		1179, 1334	3.40%	[2.6%,4.4%]	1.00		1.00		1945, 2325	
1 partner	10.8%	[7.4%,15.4%]	3.31	(1.93 - 5.67)	3.16	(1.82 - 5.50)	226, 257	8.90%	[6.7%,11.8%]	2.81	(1.87 - 4.24)	2.90	(1.84 - 4.57)	660, 547	
2+ partners	23.1%	[16.3%, 31.7%]	8.21	(4.70 - 14.34)	7.60	(4.21 - 13.72)	144, 149	15.50%	[12.3%, 19.3%]	5.28	(3.61 - 7.73)	5.45	(3.58 - 8.30)	548, 447	
**Condom-less sex with a new partner on first occasion, past year** [6]			**p<0.001**		**p<0.001**			-	-	-	-	-	-	-	-
None	4.4%	[3.3%,5.8%]	1.00		1.00		1286, 1440	-	-	-	-	-	-	-	-
At least one	16.7%	[12.5%, 21.9%]	4.41	(2.82 - 6.91)	4.09	(2.57 - 6.51)	260, 298	-	-	-	-	-	-	-	-
**Previous same-sex experience, past 5 years** [7]			**p<0.001**		**p<0.001**					**p<0.001**		**p<0.001**			**p=0.69**
No	4.9%	[3.8%,6.2%]	1.00		1.00		1453, 1721	5.30%	[4.4%,6.2%]	1.00		1.00		3071, 3256	
Yes	29.1%	[21.1%, 38.7%]	8.02	(4.88 - 13.19)	7.68	(4.68 - 12.59)	183, 114	27.90%	[19.7%, 38.0%]	7.00	(4.26 - 11.50)	6.72	(4.06 - 11.10)	126, 109	
**Pregnant in past year or currently pregnant**															-
No	-	-	-	-	-	-	-	-	-	-	-	-	-	-	-
Yes	-	-	-	-	-	-	-	-	-	-	-	-	-	-	-
**Used an STI-related service, past year**			**p<0.001**		**p<0.001**					**p<0.001**		**p<0.001**			**p=0.05**
No	3.50%	[2.6%,4.7%]	1.00		1.00		1458, 1646	2.90%	[2.2%,3.7%]	1.00		1.00		2884, 3112	
Yes	39.70%	[31.4%, 48.5%]	17.93	(11.19 - 28.71)	17.24	(10.46 - 28.42)	160, 158	47.70%	[41.4%, 54.1%]	30.96	(21.34 - 44.93)	42.92	(27.97 - 65.88)	298, 234	
**Unmet need for condoms, past year**			**p<0.001**		**p<0.001**			-	-	-	-	-	-	-	-
No	3.80	[2.8%,5.	1.00		1.00		1262,	-	-	-	-	-	-	-	-

	%	1%]					1403								
Yes	22.40 %	[17.1%, 28.6%]	7.27	(4.63 - 11.42)	6.82	(4.18 - 11.14)	237, 267	-	-	-	-	-	-	-	-

CI=confidence intervals. OR=odds ratio. aOR=age-adjusted odds ratio. PHQ-2=Patient Health Questionnaire (2 item). GAD-2=Generalized anxiety disorder (2 item)

[*] Age adjusted
[†] Men or women aged 18-44 who were sexually-experienced. Trans men and trans women are included in data for men and women, respectively. 15 women and 22 men in Natsal-COVID responded 'prefer not to say' to questions about chlamydia testing. 423 women and 229 men in Natsal-3 did not answer the question. These individuals are excluded from the denominator.
[**] Unweighted denominator <30. Results not shown due to small denominator
[***]Unweighted denominator <50. Results should be interpreted with caution due to small denominator.
[1] White includes all those who identify as White English, Welsh, Scottish, Northern Irish, British, Irish, Gypsy or Irish Traveller, or from any other White background.
[2] Mixed ethnicity includes those who identify as White and Black African, White and Black Caribbean, White and Asian or any other mixed or multiple ethnic background.
[3] Asian includes those who identify as Indian, Pakistani, Bangladeshi, Chinese or from any other Asian background
[4] Black includes those who identify as African, Caribbean, or from any other Black background.
[5] Participants were classified as having symptoms of depression or anxiety if they scored three or more on the patient health questionnaire two item (PHQ-2) or generalised anxiety disorder two item (GAD-2) scales
[6] Includes both opposite-sex and same-sex partners
[7] Same-sex experience defined as oral/anal/vaginal sex

Supplementary table 5. Variations in reporting a cervical cancer screening among eligible participants aged 25-59 years in the first year following the start of a national lockdown in Britain (23/03/2020) compared with Natsal-3 (2010-12, past three years)

	All eligible participants (Described female at birth, aged 25-59 yrs)		
	Natsal COVID (fieldwork 2021)	Natsal-3 (fieldwork 2010-12)	

	Weighted %	95% CI	OR	95% CI	aOR *	95% CI	Denominator † (unweighted, weighted)	Weighted %	95% CI	OR	95% CI	aOR *	95% CI	Denominator † (unweighted, weighted)	Interaction terms between surveys p-value
**All ages (25-59 years)**	10.3%	[9.2%,11.5%]	-	-	-	-	2949, 2837	70.6%	[70.6%,70.6%]	-	-	-	-	5176, 4770	
**Age (years)**			**p<0.001**							**p<0.001**					**p=0.01**
25-29	16.3%	[13.4%,19.8%]	2.72	(1.94 - 3.83)	-	-	582, 473	71.7%	[71.7%,71.7%]	1.29	(1.09 - 1.53)	-	-	1381, 683	
30-34	12.4%	[9.7%,15.9%]	1.98	(1.36 - 2.88)	-	-	463, 385	75.6%	[75.6%,75.6%]	1.58	(1.30 - 1.91)	-	-	1036, 656	
35-44	11.2%	[9.1%,13.8%]	1.77	(1.26 - 2.48)	-	-	816, 762	74.0%	[74.0%,74.0%]	1.45	(1.21 - 1.72)	-	-	1187, 1424	
45-59	6.7%	[5.3%,8.4%]	1.00		-	-	1088, 1217	66.3%	[66.3%,66.3%]	1.00		-	-	1572, 2007	
**Region**			**p=0.17**		**p=0.10**					**p=0.62**		**p=0.52**			**p=0.07**
England/Wales	10.6%	[9.4%,11.8%]	1.00		1.00		2720, 2599	70.5%	[70.5%,70.5%]	1.00		1.00		4740, 4333	
Scotland	7.5%	[4.6%,12.0%]	0.68	(0.40 - 1.17)	0.64	(0.38 - 1.09)	229, 238	71.8%	[71.8%,71.8%]	1.07	(0.83 - 1.38)	1.09	(0.84 - 1.40)	436, 437	
**Rurality**			**p=0.02**		**p=0.03**			-	-	-	-	-	-	-	-
Urban	10.7%	[9.4%,12.2%]	1.00		1.00		2117, 2047	-	-	-	-	-	-	-	-
Rural	6.9%	[4.9%,9.8%]	0.62	(0.42 - 0.93)	0.65	(0.43 - 0.97)	435, 408	-	-	-	-	-	-	-	-

**Ethnicity**			**p=0.003**		**p=0.01**					**p=0.30**		**p=0.12**			**p=0.10**
White [1]	9.9%	[8.8%,11.2%]	1.00		1.00		2644, 2475	71.1%	[71.1%,71.1%]	1.00		1.00		4527, 4160	
Mixed, multiple, or other [2]	29.0%	[17.2%,44.5%]	3.70	(1.86 - 7.34)	3.26	(1.58 - 6.73)	79, 66	72.6%	[72.6%,72.6%]	1.08	(0.69 - 1.69)	1.03	(0.65 - 1.61)	141, 112	
Asian or Asian British [3]	10.4%	[6.3%,16.5%]	1.05	(0.60 - 1.82)	0.92	(0.52 - 1.63)	151, 188	66.5%	[66.5%,66.5%]	0.81	(0.62 - 1.05)	0.74	(0.56 - 0.96)	304, 297	
Black or Black British [4]	9.0%	[3.5%,21.1%]	0.90	(0.33 - 2.45)	0.75	(0.28 - 2.05)	52, 73	66.9%	[66.9%,66.9%]	0.82	(0.56 - 1.20)	0.81	(0.56 - 1.18)	194, 191	
**Sexual identity**			**p=0.001**		**p=0.003**					**p=0.93**		**p=0.86**			**p=0.01**
Heterosexual/straight	10.2%	[9.1%,11.5%]	1.00		1.00		2663, 2706	70.7%	[70.7%,70.7%]	1.00		1.00		4994, 4626	
Gay or lesbian	3.3%	[1.2%,8.6%]	0.30	(0.11 - 0.83)	0.23	(0.08 - 0.67)	68, 31	66.3%	[66.3%,66.3%]	0.82	(0.44 - 1.51)	0.79	(0.43 - 1.46)	71, 60	
Bisexual	21.7%	[13.9%,32.3%]	2.43	(1.39 - 4.24)	1.63	(0.97 - 2.74)	147, 40	70.7%	[70.7%,70.7%]	1.00	(0.58 - 1.73)	0.89	(0.51 - 1.56)	87, 61	
Other	5.3%	[1.1%,21.7%]	0.49	(0.10 - 2.44)	0.24	(0.05 - 1.20)	43, 27 ***	-	-	-	-	-	-	14, 13 **	
**Social grade**			**p=0.87**		**p=0.83**			-	-	-	-	-	-	-	-
AB Higher and intermediate managerial/administrative/professional occupation	9.8%	[7.8%,12.2%]	1.00		1.00		742, 660	-	-	-	-	-	-	-	-
C1 Supervisory, clerical and junior managerial/administrative/professional occupations/C2 Skilled manual occupations	10.5%	[9.0%,12.2%]	1.09	(0.80 - 1.47)	1.10	(0.81 - 1.50)	1553, 1554	-	-	-	-	-	-	-	-
D Semi-skilled and unskilled manual occupations/E On state benefit, unemployed and lowest grade occupations	10.3%	[8.2%,13.0%]	1.07	(0.74 - 1.53)	1.06	(0.73 - 1.53)	654, 623	-	-	-	-	-	-	-	-
**Highest education qualification**			**p=0.03**		**p=0.16**					**p<0.001**		**p<0.001**			**p=0.92**

Degree	11.8%	[10.2%,13.7%]	1.00		1.00		1488, 1393	75.00%	[75.0%,75.0%]	1.00		1.00		1629, 1486	
Below degree	8.9%	[7.5%,10.6%]	0.73	(0.56 - 0.94)	0.81	(0.63 - 1.05)	1346, 1331	69.90%	[69.9%,69.9%]	0.77	(0.66 - 0.90)	0.81	(0.70 - 0.95)	3070, 2838	
No qual	7.5%	[3.9%,14.0%]	0.60	(0.29 - 1.23)	0.62	(0.30 - 1.27)	115, 114	60.60%	[60.6%,60.6%]	0.51	(0.40 - 0.66)	0.58	(0.45 - 0.75)	469, 438	
**Born outside the UK**			**p<0.001**		**p=0.001**			-	-	-	-	-	-	-	-
No	9.3%	[8.2%,10.5%]	1.00		1.00		2579, 2456	-	-	-	-	-	-	-	-
Yes	17.5%	[13.5%,22.3%]	2.07	(1.48 - 2.90)	1.78	(1.26 - 2.51)	349, 358	-	-	-	-	-	-	-	-
**Relationship status**			**p=0.05**		**p=0.15**					**p<0.001**		**p<0.001**			**p=0.27**
Married/steady and living together	9.9%	[8.7%,11.4%]	1.00		1.00		1917, 1871	71.7%	[71.7%,71.7%]	1.00		1.00		3204, 3465	
Steady not living together	15.4%	[11.0%,21.0%]	1.64	(1.09 - 2.48)	1.47	(0.97 - 2.25)	227, 202	75.8%	[75.8%,75.8%]	1.24	(0.97 - 1.58)	1.18	(0.92 - 1.50)	653, 409	
Not in a steady relationship	10.0%	[7.9%,12.5%]	1.01	(0.75 - 1.35)	0.95	(0.70 - 1.28)	797, 754	64.7%	[64.7%,64.7%]	0.72	(0.62 - 0.84)	0.72	(0.62 - 0.85)	1287, 873	
**Days drinking, past 7 days**			**p=0.11**		**p=0.09**			-	-	-	-	-	-	-	-
0 days	9.3%	[7.8%,11.0%]	1.00		1.00		1342, 1302	-	-	-	-	-	-	-	-
1-2 days	11.9%	[10.0%,14.2%]	1.33	(1.00 - 1.75)	1.31	(0.99 - 1.73)	1019, 972	-	-	-	-	-	-	-	-
3-4 days	8.6%	[6.1%,12.0%]	0.92	(0.60 - 1.41)	0.92	(0.60 - 1.42)	358, 346	-	-	-	-	-	-	-	-
5-7 days	12.4%	[8.6%,17.7%]	1.39	(0.88 - 2.19)	1.53	(0.96 - 2.42)	222, 208	-	-	-	-	-	-	-	-
**Currently smoker**			**p=0.003**		**p=0.02**					**p<0.001**		**p<0.001**			**p=0.0003**
No	9.4%	[8.3%,10.7%]	1.00		1.00		2383, 2296	72.4%	[72.4%,72.4%]	1.00		1.00		3756, 3622	

Yes	14.0%	[11.2%,17.4%]	1.57	(1.17 - 2.10)	1.42	(1.05 - 1.92)	557, 532	64.9%	[64.9%,64.9%]	0.70	(0.61 - 0.82)	0.68	(0.59 - 0.79)	1420, 1148	
**Importance of sexual health, past year**			**p<0.001**		**p<0.001**			-	-	-	-	-	-	-	-
Very important/somewhat important	13.3%	[11.6%,15.2%]	1.00		1.00		1520, 1428	-	-	-	-	-	-	-	-
Not very important/not important	8.3%	[6.7%,10.2%]	0.59	(0.45 - 0.78)	0.67	(0.51 - 0.89)	1050, 1032	-	-	-	-	-	-	-	-
This does not apply to me	4.0%	[2.2%,7.0%]	0.27	(0.14 - 0.50)	0.30	(0.16 - 0.56)	303, 298	-	-	-	-	-	-	-	-
**Symptoms of depression (PHQ-2)** [5]			**p=0.21**		**p=0.93**					**p=0.02**		**p=0.03**			**p=0.99**
No	9.8%	[8.6%,11.2%]	1.00		1.00		2033, 1983	71.3%	[71.3%,71.3%]	1.00		1.00		4535, 4210	
Yes	11.4%	[9.4%,13.8%]	1.18	(0.91 - 1.55)	1.01	(0.77 - 1.34)	885, 826	65.9%	[65.9%,65.9%]	0.78	(0.63 - 0.96)	0.80	(0.64 - 0.98)	629, 546	
**Symptoms of anxiety (GAD-2)** [5]			**p=0.07**		**p=0.54**			-	-	-	-	-	-	-	-
No	9.6%	[8.4%,11.1%]	1.00		1.00		1976, 1955	-	-	-	-	-	-	-	-
Yes	11.9%	[9.9%,14.2%]	1.26	(0.98 - 1.64)	1.09	(0.84 - 1.41)	956, 866	-	-	-	-	-	-	-	-
**Total sexual partners, past year** [6]			**p<0.001**		**p=0.006**					**p<0.001**		**p<0.001**			**p=0.01**
0 partners	8.4%	[6.5%,10.6%]	1.00		1.00		873, 860	58.0%	[58.0%,58.0%]	1.00		1.00		634, 543	
1 partner	10.8%	[9.4%,12.4%]	1.33	(0.98 - 1.80)	1.28	(0.94 - 1.75)	1792, 1704	72.3%	[72.3%,72.3%]	1.89	(1.56 - 2.29)	1.72	(1.41 - 2.09)	3884, 3762	
2+ partners	24.4%	[16.8%,34.2%]	3.55	(2.06 - 6.10)	2.48	(1.42 - 4.33)	114, 99	73.1%	[73.1%,73.1%]	1.97	(1.47 - 2.63)	1.64	(1.22 - 2.21)	579, 385	
**New sexual partners, past year** [6]			**p<0.001**		**p=0.002**					**p=0.12**		**p=0.22**			**p=0.06**

0 partners	9.9%	[8.8%,11.2%]	1.00		1.00		2560, 2469	70.5%	[70.5%,70.5%]	1.00		1.00		4211, 4089	
1 partner	13.9%	[9.1%,20.7%]	1.47	(0.89 - 2.42)	1.20	(0.73 - 1.97)	163, 148	69.5%	[69.5%,69.5%]	0.95	(0.76 - 1.19)	0.88	(0.70 - 1.10)	620, 435	
2+ partners	32.3%	[19.7%,48.1%]	4.33	(2.20 - 8.55)	3.29	(1.69 - 6.40)	51, 43	77.5%	[77.5%,77.5%]	1.44	(1.00 - 2.06)	1.24	(0.87 - 1.78)	261, 160	
**Condom-less sex with a new partner on first occasion, past year** [6]			**p<0.001**		**p=0.01**			-	-	-	-	-	-	-	-
None	10.0%	[8.9%,11.3%]	1.00		1.00		2658, 2550	-	-	-	-	-	-	-	-
At least one	20.5%	[13.9%,29.1%]	2.31	(1.42 - 3.75)	1.86	(1.14 - 3.03)	131, 120	-	-	-	-	-	-	-	-
**Previous same-sex experience, past 5 years** [7]			**p=0.41**		**p=0.26**					**p=0.56**		**p=0.86**			**p=0.21**
No	10.4%	[9.3%,11.7%]	1.00		1.00		2761, 2707	70.5%	[70.5%,70.5%]	1.00		1.00		4965, 4606	
Yes	8.1%	[4.4%,14.4%]	0.76	(0.39 - 1.47)	0.68	(0.35 - 1.32)	127, 65	72.8%	[72.8%,72.8%]	1.12	(0.77 - 1.61)	1.03	(0.71 - 1.50)	209, 161	
**Chlamydia test, past year**			**p<0.001**		**p<0.001**					**p<0.001**		**p<0.001**			**p<0.001**
No/not sure	9.3%	[8.3%,10.5%]	1.00		1.00		2820, 2724	72.7%	[72.7%,72.7%]	1.00		1.00		2840, 2277	
Yes	40.6%	[30.8%,51.2%]	6.62	(4.23 - 10.36)	2.65	(1.64 - 4.28)	105, 89	81.5%	[81.5%,81.5%]	1.65	(1.29 - 2.12)	1.68	(1.30 - 2.18)	702, 433	
**HIV test, past year**			**p<0.001**		**p<0.001**					**p=0.28**		**p=0.79**			**p<0.001**
No/not sure	9.5%	[8.5%,10.7%]	1.00		1.00		2783, 2680	70.4%	[70.4%,70.4%]	1.00		1.00		4476, 4214	
Yes	26.3%	[18.8%,35.5%]	3.38	(2.15 - 5.31)	5.05	(3.19 - 7.99)	145, 135	73.7%	[73.7%,73.7%]	1.18	(0.87 - 1.60)	0.96	(0.71 - 1.31)	361, 243	
**Used an STI-related service, past year**			**p<0.001**		**p<0.001**					**p=0.001**		**p=0.02**			**p=0.002**

No	9.2%	[8.2%,10.4%]	1.00		1.00		2830, 2730	70.6%	[70.6%,70.6%]	1.00		1.00		4856, 4542	
Yes	38.0%	[28.9%,48.1%]	6.04	(3.92 - 9.30)	4.74	(3.02 - 7.44)	119, 107	82.9%	[82.9%,82.9%]	2.01	(1.32 - 3.06)	1.65	(1.08 - 2.53)	227, 141	
**Unmet need for condoms, past year**			**p=0.20**		**p=0.95**			-	-	-	-	-	-	-	-
No	10.70%	[9.5%,12.0%]	1.00		1.00		2525, 2410	-	-	-	-	-	-	-	-
Yes	15.10%	[8.8%,24.7%]	1.50	(0.80 - 2.80)	1.02	(0.53 - 1.96)		-	-	-	-	-	-	-	-

CI=confidence intervals. OR=odds ratio. aOR=age-adjusted odds ratio. PHQ-2=Patient Health Questionnaire (2 item). GAD-2=Generalized anxiety disorder (2 item)

[*] Age adjusted

[†] Participants described female at birth aged 25-29. 90 eligible participants responded 'prefer not to say' to questions about cervical cancer screening. 144 in Natsal-3 did not answer the question. These individuals are excluded from the denominator.

[**] Unweighted denominator <30. Results not shown due to small denominator

[***]Unweighted denominator <50. Results should be interpreted with caution due to small denominator.

[1] White includes all those who identify as White English, Welsh, Scottish, Northern Irish, British, Irish, Gypsy or Irish Traveller, or from any other White background.

[2] Mixed ethnicity includes those who identify as White and Black African, White and Black Caribbean, White and Asian or any other mixed or multiple ethnic background.

[3] Asian includes those who identify as Indian, Pakistani, Bangladeshi, Chinese or from any other Asian background

[4] Black includes those who identify as African, Caribbean, or from any other Black background.

[5] Participants were classified as having symptoms of depression or anxiety if they scored three or more on the patient health questionnaire two item (PHQ-2) or generalised anxiety disorder two item (GAD-2) scales

[6] Includes both opposite-sex and same-sex partners

[7] Same-sex experience defined as oral/anal/vaginal sex

All percentages are weighted. These are row percentages which describe reported use of cervical cancer screening in the past year (Natsal-COVID) or past three years (Natsal-3) within certain subgroups.
